# The mechanism of ϕC31 integrase directionality: experimental analysis and computational modelling

**DOI:** 10.1093/nar/gkw616

**Published:** 2016-07-07

**Authors:** Alexandra Pokhilko, Jia Zhao, Oliver Ebenhöh, Margaret C. M. Smith, W. Marshall Stark, Sean D. Colloms

**Affiliations:** 1 Institute of Molecular, Cell and Systems Biology, Bower Building, University of Glasgow, Glasgow G12 8QQ, Scotland, UK; 2 Cluster of Excellence on Plant Sciences (CEPLAS), Heinrich-Heine-University, Universitätsstraße 1, D-40225 Düsseldorf, Germany; 3 Institute for Complex Systems and Mathematical Biology, University of Aberdeen, AB24 3UE, UK; 4 Department of Biology, University of York, Wentworth Way, York YO10 5DD, UK

## Abstract

Serine integrases, DNA site-specific recombinases used by bacteriophages for integration and excision of their DNA to and from their host genomes, are increasingly being used as tools for programmed rearrangements of DNA molecules for biotechnology and synthetic biology. A useful feature of serine integrases is the simple regulation and unidirectionality of their reactions. Recombination between the phage *attP* and host *attB* sites is promoted by the serine integrase alone, giving recombinant *attL* and *attR* sites, whereas the ‘reverse’ reaction (between *attL* and *attR*) requires an additional protein, the recombination directionality factor (RDF). Here, we present new experimental data on the kinetics and regulation of recombination reactions mediated by ϕC31 integrase and its RDF, and use these data as the basis for a mathematical model of the reactions. The model accounts for the unidirectionality of the *attP* × *attB* and *attL* × *attR* reactions by hypothesizing the formation of structurally distinct, kinetically stable integrase–DNA product complexes, dependent on the presence or absence of RDF. The model accounts for all the available experimental data, and predicts how mutations of the proteins or alterations of reaction conditions might increase the conversion efficiency of recombination.

## INTRODUCTION

The serine integrases are a group of DNA site-specific recombinases whose natural functions are to integrate and excise bacteriophage DNA into and out from the host bacterial genomic DNA (1). The serine integrases have recently received much attention as potential tools for experimental genetic manipulation, biotechnology and synthetic biology, and many examples have been characterized *in vitro* and *in vivo* (1,2). The subject of this report is ϕC31 integrase, perhaps the most extensively studied member of the group, which has been exploited for applications including integrating vectors for bacteria, gene therapy in mammalian cells, gene knock-in/knock-out in various experimental organisms, gene/metabolic pathway assembly, genetic switches, logic gates and memory devices (2–9).

**Figure 1.**
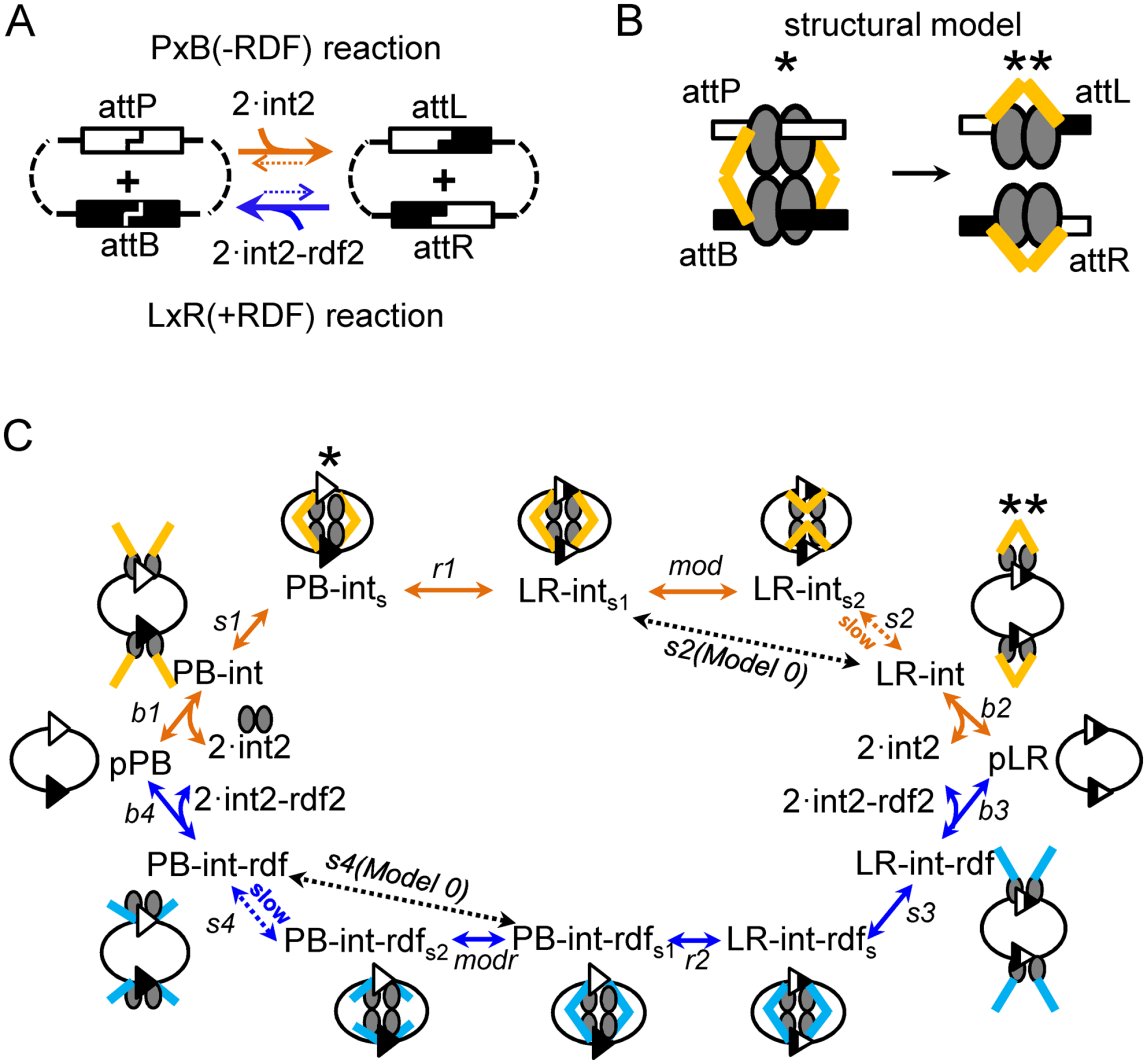
Site-specific recombination by ϕC31 integrase: mechanisms and models. (**A**) Directionality of integrase-catalysed reactions. The ‘forward’ (P × B) reaction (orange arrows) is promoted by integrase (initiated by binding of integrase dimers (int2) to each *att* site). The ‘reverse’ (L × R) reaction is not observed in the absence of the recombination directionality factor (RDF). In the presence of RDF, which interacts with integrase dimers, the L × R reaction (blue arrows) is favoured. (**B**) Cartoons showing a structure-based hypothesis (14) for substrate and product synaptic complexes of a P × B (−RDF) reaction, illustrating proposed interactions of the integrase coiled-coil (CC) domains, either between subunits bound to different *att* sites (left), or between two subunits bound on a single *att* site (right). Integrase monomers are shown as grey ovals, with the coiled coil domains as yellow sticks. (**C**) Scheme illustrating the models described in this work (see text for details). The upper pathway (orange arrows) is for the P × B (−RDF) reaction; the lower pathway (blue arrows) is for the L × R (+RDF) reaction. The intermediate names are in plain text, and cartoons above and below show their hypothesized structures. Note that in the L × R (+RDF) pathway, RDF is not shown in the cartoons, but the sticks representing the CC domains (which might interact with RDF) are in blue instead of yellow. Reaction steps (indicated by arrows) are named in italics. Corresponding complexes in parts B and C are indicated by asterisks (*, **).

In common with other serine integrase systems, ϕC31 integrase catalyses recombination between non-identical short (40–50 bp) DNA sites that each bind an integrase dimer. Recombination between the phage-derived *attP* site and the bacterial *attB* site (P × B) is unidirectional, forming two recombinant sites *attL* and *attR*, each of which comprises an *attP* ‘half-site’ joined to an *attB* ‘half-site’ (Figure 1A). The ‘reverse’ reaction between *attL* and *attR* (L × R) is not observed in the presence of the integrase alone. However, the presence of a recombination directionality factor (RDF) protein transforms the activity of integrase so that it promotes L × R recombination, leading to *attP* and *attB* products (1,10–13).

The molecular basis for this remarkable directionality is not well understood. It is not accounted for by differences between the free energies of unbound substrate and product DNA molecules (for reactions between linear substrates, the substrate and product molecules are expected to be isoenergetic), nor is the reaction coupled to any other chemical transformation (such as adenosine triphosphate hydrolysis). It seems therefore that the reactions must be driven by the formation of stable (energetically favourable) protein–DNA complexes as end-products. The mode of action of the RDF also remains mysterious. The RDF is known to interact directly with integrase (12,13); somehow these integrase–RDF interactions promote the L × R reaction and suppress the P × B reaction. Recent crystallographic structures of a serine integrase bound to its recombination site DNA have led to a testable model for the structural basis of directional recombination and the role of the RDF (1,14–16), though the site of interaction with the RDF has not been established and there are as yet no high-resolution RDF structures (1,14–16).

Mathematical modelling can be used to explore regulatory mechanisms in biological systems (17), and may help us to understand the factors determining the directionality of reactions promoted by ϕC31 integrase and other serine integrases. This is important not only from a fundamental point of view, but also to provide a knowledge base for practical applications of these systems. Here, we have built a quantitative and thermodynamically consistent mathematical model of the reactions of ϕC31 integrase in the absence or presence of its RDF, deriving the parameters from previous experimental analyses and our new data. The model reveals that certain features of the system, including the formation of kinetically stable DNA–integrase and DNA–integrase–RDF complexes, might play key roles in reaction directionality.

## MATERIALS AND METHODS

### Experimental methods

#### Bacterial strain, plasmids and oligonucleotides

**Figure 2.**
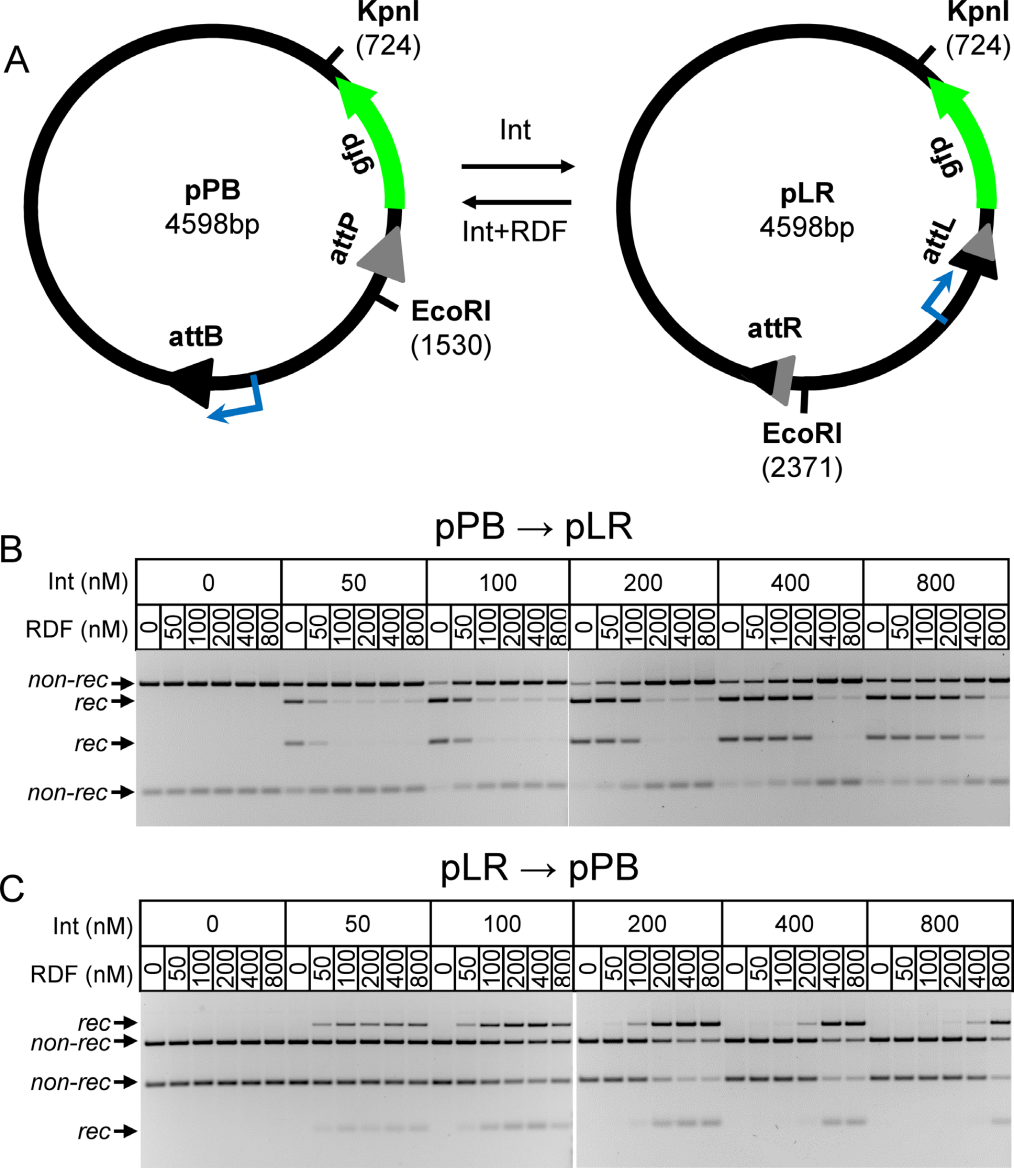
Experimental analysis of ϕC31 integrase-mediated recombination *in vitro*. (**A**) The substrate plasmids. Note that pLR is the recombination product of pPB, and *vice versa*. The promoter for GFP is shown in blue. The numbers in brackets indicate the map positions of the restriction sites used in the experiments. (**B** and **C**) Representative recombination assays with pPB (B) and pLR (C); effects of protein concentration. Concentrations of ϕC31 integrase (Int) and RDF were as indicated above each lane. Plasmid concentration was 10 nM. Reactions were incubated for 3 h. Reaction products were digested with KpnI and EcoRI, and analysed by agarose gel electrophoresis as described in ‘Materials and Methods’ section. DNA restriction fragments are indicated as non-recombinant substrate (non-rec) and recombinant product (rec).

Substrate plasmid DNA used for *in vitro* assays was prepared from the *Escherichia coli* strain DH5 (F^−^ λ^–^ Δ(*lacZYA-argF*)U169 *recA1 endA1 hsdR17* (r_K_^–^, m_K_^+^) *phoA supE44 thi*-1 *gyrA96 relA1*) using a plasmid miniprep kit (Qiagen) according to the manufacturer's instructions, with one extra wash step with buffer PE. DNA concentrations were determined by measuring the absorbance at 260 nm (absorbance of 1, 1 cm pathlength, estimated as 50 μg/ml). Substrate plasmids pPB and pLR (Figure 2A), containing pairs of *att* sites (*attB, attP* and *attR, attL* respectively) in inverted repeat (separated by 899 bp centre to centre) were constructed in the pMK-RQ plasmid backbone (GeneArt). The invertible DNA segment contains a constitutive promoter, which initiates the transcription of a *gfp* gene when the segment is in one orientation but not the other. Full sequences of these plasmids are available on request. Oligodeoxynucleotides for assembly of fluorescently labelled linear *attB* or *attP* DNA fragments were synthesized by Integrated DNA Technologies (IDT). The sequences of the oligodeoxynucleotides are shown in Supplementary Text S1.1.1. The oligodeoxynucleotides were dissolved at 100 μM in TE_0.1_ buffer (10 mM Tris–HCl, 0.1 mM ethylenediaminetetraacetic acid (EDTA), pH 8.0). Fluorescent labelled oligonucleotides (10 μM 5′-FAM-*attP*-bot or 5′-Cy5-*attB*-bot) were mixed with an excess of the corresponding unlabelled oligonucleotide (10.2 μM *attP*-top or *attB*-top respectively) in TE_0.1_ buffer. The mixtures were heated to 87°C for 5 min and cooled down to 25°C over 2 h to anneal the two strands. Recombination converts the 79 bp FAM-labelled *attP* and 85 bp Cy5-labelled *attB* to 65 bp FAM-labelled *attL* and 99 bp Cy5-labelled *attR* fragments.

#### Proteins

Proteins (ϕC31 integrase and its RDF, known as gp3) were purified as previously described (11,12,18). Purity and concentration of the proteins were estimated by sodium dodecyl sulphate-polyacrylamide gel electrophoresis and measurement of absorbance at 280 nm, assuming a calculated extinction coefficient at 280 nm of 78 000 l mol^−1^ cm^−1^ for integrase and 43 000 l mol^−1^ cm^−1^ for gp3. All protein concentrations given in this paper refer to monomer. Both proteins were diluted in protein dilution buffer containing 25 mM Tris·HCl (pH 7.5), 1 mM DTT, 1 M NaCl and 50% (vol/vol) glycerol, and stored at −20°C.

#### In vitro recombination assays

Aliquots of ϕC31 integrase or integrase premixed with RDF were prepared in protein dilution buffer at 10 times their desired final concentrations. Reactions (20 μl for the 3 h end-point measurements or 130 μl for the time courses) were set up by pre-incubating 10 nM plasmid substrate in a buffer containing 25 mM Tris·HCl pH 7.5, 2.5 mM spermidine, 25 mM NaCl and 50 μg/ml bovine serum albumin (BSA) at 30°C for 10 min. Recombination was then started by adding protein solution (1/10 of final volume in protein dilution buffer, so that the final reaction buffer contained 125 mM NaCl), and reactions were incubated at 30°C. For the time courses, 10 μl aliquots were taken and stopped at each time point. Other reactions were for 3 h at 30°C. For all reactions, recombination was stopped by heating the samples at 80°C for 10 min. For reactions of linear DNA substrates, annealed oligonucleotide *attP* and *attB* (10 nM of each) were added instead of plasmid.

Reactions of plasmid substrates were analysed by restriction enzyme digestion followed by gel electrophoresis. After digestion with restriction enzymes EcoRI and KpnI at 37°C for 3 h, 5 μl of loading buffer (25 mM Tris·HCl (pH 8.2), 20% (w/v) Ficoll, 0.5% sodium dodecyl sulphate, 5 mg/ml protease K, 0.25 mg/ml bromophenol blue) was added to each sample, and the mixture was incubated at 37°C for 30 min prior to loading onto a 1.2% (wt/vol) agarose gel (24 × 16 × 0.6 cm) in Tris-Acetate EDTA (TAE) buffer (40 mM Tris base, 25 mM acetic acid, 1 mM EDTA and 20 mM sodium acetate). Gels were run for 16 h at 0.65 V/cm, stained extensively (>30 min) with ethidium bromide (0.5 μg/ml), destained in TAE buffer for 10 min and imaged using a Bio-Rad GelDoc UV Transilluminator. The intensities of DNA bands on the gel were quantitated using the volume analysis tool of Quantity One software (Biorad) using background rectangle subtraction, and the proportions of recombinant products were determined after correcting the intensity values for fragment size. The accuracy of quantitation by this method was confirmed by introducing product DNA into chemically competent *E. coli* cells and counting the proportion of transformant colonies that expressed GFP (data not shown). All experiments were carried out in triplicate. Average values and standard deviations are shown on all experimental figures.

Recombination of linear substrates was assayed by running 8% polyacrylamide gels (37.5:1 acrylamide:bisacrylamide) in Tris/Borate/EDTA (TBE) buffer (90 mM Tris base, 90 mM boric acid and 2 mM EDTA). Gels were scanned using a GE Healthcare Typhoon FLA9500 laser scanner in fluorescence mode set to detect FAM (473 nm laser and 510 nm long pass LPB filter) and Cy5 (635 nm laser and 665 nm long pass LPR filter). The fluorescent intensities of linear DNA bands on the gel were quantitated using the volume analysis tool of Quantity One software (Biorad).

### Modelling

Our modelling uses ordinary differential equations (ODEs) to describe the kinetics of the reaction systems (17). The model is based on the reaction scheme of Figure 1C, which describes interconversions of different complexes of integrase and RDF with DNA substrates and products. In the Results section we describe a simplified version of the model (Model 0) and the final model (Model 1), which differ by the presence of conformational changes in synaptic complexes and slow steps of dissociation of product synapses (Figure 1C). Throughout the manuscript we use the abbreviations P × B and L × R to refer to the *attP* × *attB* and *attL* × *attR* reactions respectively, and the abbreviations pPB and pLR to refer to the corresponding plasmid substrates. We also developed a version of Model 1 for intermolecular recombination of linear DNA substrates (Supplementary Figure S1) described in the relevant Results section. ODEs of all models and parameter values are presented in Supplementary Text S1.2 and Supplementary Tables S1–3.

**Figure 3.**
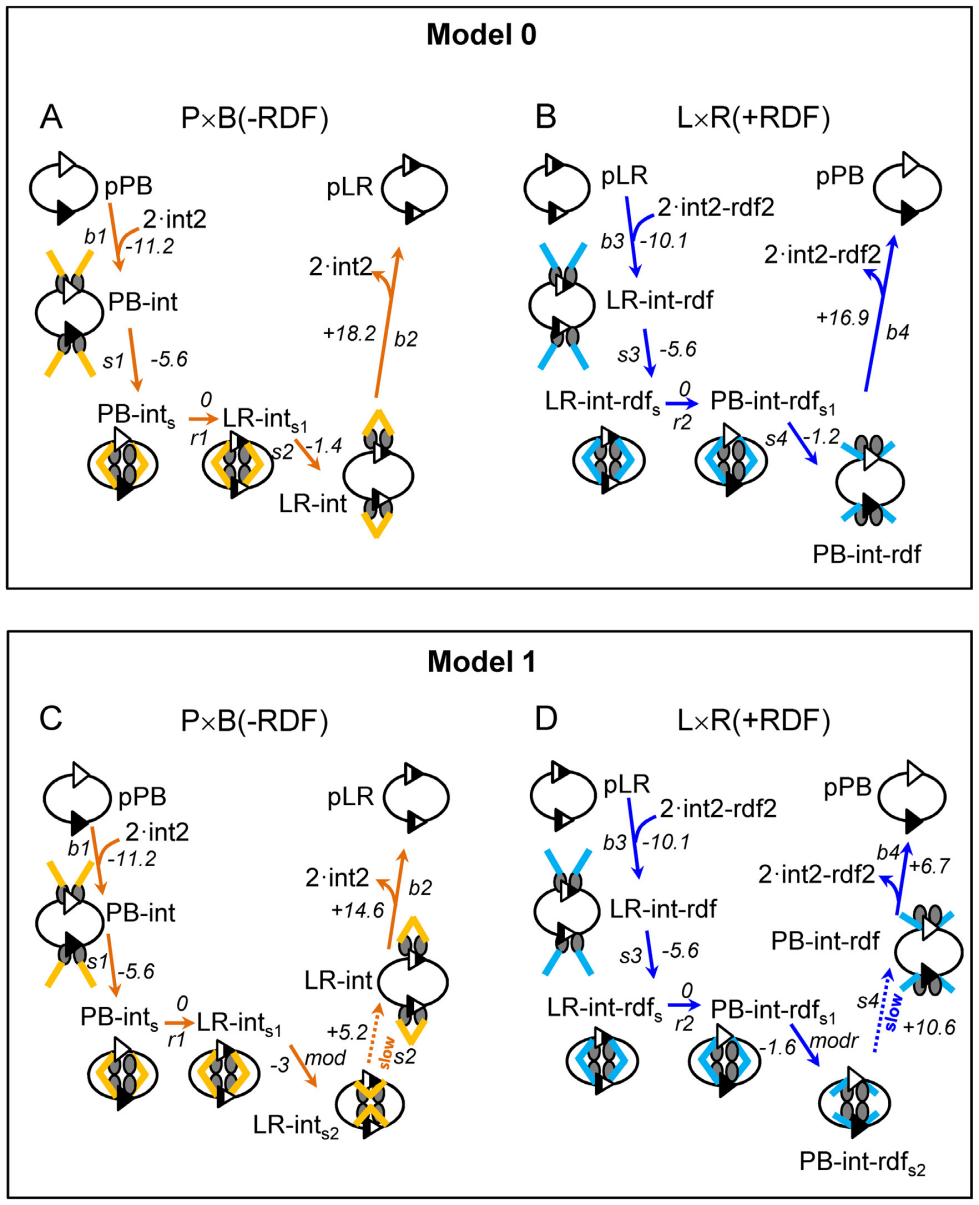
Energetic landscapes of integrase reactions in Model 0 and Model 1. Names of reaction steps/species, cartoons and colour coding of arrows are as in Figure 1C. Gibbs free energy changes (ΔG; numbers in italics, in kJ/mol) for individual reaction steps were calculated using concentrations of 10 nM (for DNA-containing species) and 200 nM (for unbound protein complexes int2 or int2–rdf2). The orientation of the arrows (up, down, across) corresponds to the sign of ΔG (−, +, 0). (**A**) P × B (−RDF) (Model 0). (**B**) L × R (+RDF) (Model 0). (**C**) P × B (−RDF) (Model 1). (**D**) L × R (+RDF) (Model 1). See text for further details.

All reaction steps of our models are assumed to be reversible and described by first or second order kinetics. Therefore, each reaction step is characterized by two parameters; the rate constants of forward and reverse reactions, with the equilibrium constant for each step defined as the ratio of forward to reverse rate constants. Equilibrium constants are named according to the steps that they represent (Figure 1C): integrase or integrase–RDF binding to the DNA, which are called ‘b’ steps, with equilibrium constants *K*_b1_, *K*_b2_, *K*_b3_, *K*_b4_; pairing of two DNA-bound integrase dimers or integrase–RDF complexes to form a synapse (synapsis), which are called ‘s’ steps, with dimensionless (for models of plasmid recombination) equilibrium constants *K*_s1_, *K*_s2_, *K*_s3_, *K*_s4_; and recombination steps called ‘r’ with the equilibrium constants called *K*_r1_, *K*_r2_. Additionally, Model 1 has two steps (‘mod’ and ‘modr’) corresponding to conformational changes (modification) of synaptic complexes, with equilibrium constants *K*_mod_, *K*_modr_. The motivation for including these modification steps is described in the Results section. Interactions between integrase and RDF in solution were modelled with dissociation constants *K*_ii_ for integrase dimerization and *K*_ir_ for binding of integrase to RDF (Supplementary Figure S2A and B; Supplementary Text S1.2). In addition, the observed inhibition of the P × B reaction at high integrase concentrations was modelled through the formation of non-functional multimeric complexes of integrase with PB substrate (Supplementary Figure S2C). The 25 Model 1 parameters were fitted to our experimental data (presented in Supplementary Table S4), which contains 92 data points including time courses and 3-h end-point measurements under different integrase and RDF concentrations. Different parameters are constrained by different aspects of the data. Some representative examples of the effects of parameters on the reaction outcomes are shown in Supplementary Figure S3. Supplementary Text S1.2.1 gives further details on the relationships between the parameters and the modelled outcomes. Briefly, the affinities of binding of integrase dimers and integrase–RDF complexes to the DNA are constrained by the dependence of reactions on integrase and RDF concentrations (Supplementary Table S4; also, affinities measured from binding gels (19)). The parameters for the formation of substrate synapses are constrained by observed reaction rates. The rate constants of protein and DNA interactions were chosen to be within a typically observed range (20,21). The whole set of parameters is additionally constrained by energy conservation equations as discussed in Results (Figure 3). The change in the free energy of each reaction step ΔG was calculated as ΔG = RT·ln(Γ/*K*_eq_), using concentrations of 10 nM for DNA-containing species and 200 nM for unbound protein complexes, where Γ is the mass action ratio and *K*_eq_ is the equilibrium constant of the corresponding reaction.

A 2-fold (100%) change in any parameter of Model 1 causes <33% change in the maximal yields of reaction products (Supplementary Figure S4). The model is thus very robust to parameter perturbations.

The system of ODEs was solved using MATLAB, integrated with the stiff solver ode15s (The MathWorks UK, Cambridge). The MATLAB code of the model is provided in Supplementary Text S1.4 and is freely available at https://github.com/QTB-HHU/integraseModel.

## RESULTS

### *In vitro* recombination assays

#### Description of the system

Published data on the kinetics of ϕC31 integrase-mediated reactions have been obtained under a variety of experimental conditions, and using different kinds of DNA substrates. Intramolecular reactions between sites on supercoiled plasmids, intermolecular reactions between two linear molecules and intermolecular reactions between linear and circular plasmid molecules have been reported (10–12,18,19,22). The maximal proportions of reaction products (reaction yield) and reaction rates vary between these different experimental protocols (10–12,18,19,22), likely because of variations in a number of factors including DNA substrate structure, buffer conditions, protein and DNA concentrations, protein and DNA purity, and level of plasmid DNA supercoiling. Also, ratios of *attP* to *attB* (or *attL* to *attR*) sites have been adjusted in some experiments to maximize recombination of one of the substrates (11,22). It is thus difficult to build a model based on the existing data from these various sources. To overcome this problem, we created a new dataset by systematically measuring the kinetics of ϕC31 integrase-mediated intramolecular recombination of supercoiled plasmid substrates under defined conditions *in vitro*. The *attP* × *attB* (pPB) and *attL* × *attR* (pLR) plasmid substrates both have two *att* sites in inverted repeat and are identical except for the *att* sites and the orientation of the DNA segment between the two sites (the pLR substrate is the recombination product of the pPB substrate and *vice versa*) (Figure 2A). Use of substrates with *att* sites in inverted repeat (‘head-to-head’), avoids the potential bias due to entropy changes that might be introduced in reactions that change DNA geometry (such as intramolecular reaction between sites in direct repeat on a circular plasmid, which leads to two separate product DNA circles). For comparison, we also conducted experiments to assay intermolecular recombination between *attB* and *attP* sites located on separate linear (oligonucleotide) DNA molecules.

#### Reaction kinetics with plasmid substrates, and dependence of reactions on integrase and RDF concentrations

**Figure 4.**
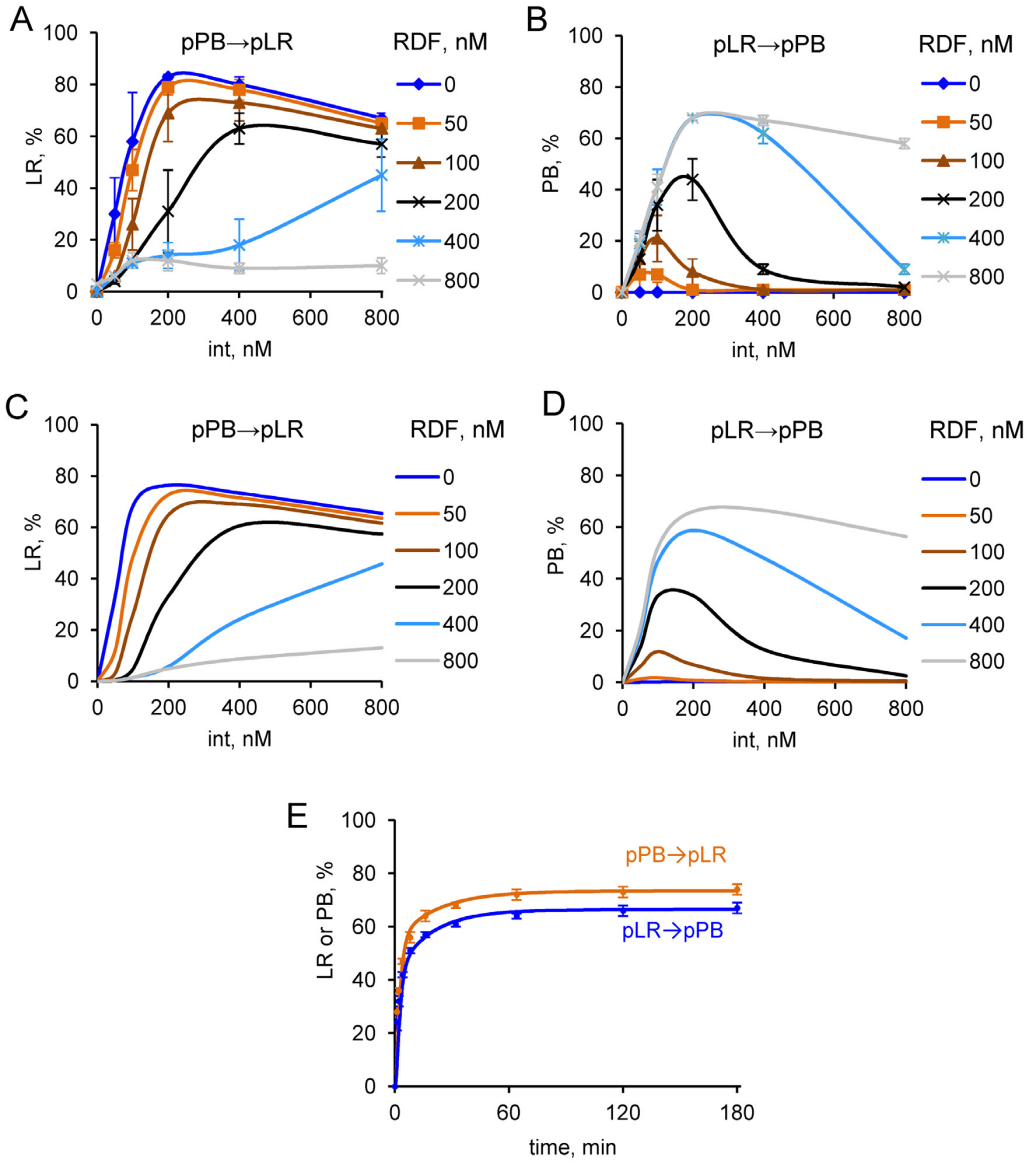
Experimentally determined and modelled kinetics and protein concentration dependence of ϕC31 integrase-mediated recombination. (**A–D**) The dependence of recombination on integrase and RDF concentration in the experiments (A and B) and in Model 1 (C and D). Extent of recombination after 3 h was measured experimentally (A and B) or calculated using Model 1 (C and D) for the P × B (−RDF) reaction (A and C) and the L × R (+RDF) reaction (B and D), with the indicated integrase and RDF concentrations (plasmid substrate concentration 10 nM). (**E**) Time-courses of P × B (−RDF) and L × R (+RDF) reactions (orange and blue respectively). Reactions were with 10 nM plasmid substrate (pPB or pLR) and 400 nM integrase, and 800 nM RDF for the L × R (+RDF) reaction. The experimental data are shown by symbols and simulated kinetics (Model 1) by lines. Data points on A, B and E are shown as mean and standard deviation from three independent replicates of the experiments.

First, we measured the effects of varying integrase and RDF concentrations on the extent of recombination after 3 h (Figure 2B and C; quantitative data in Figure 4A and B). The extent of *attP* × *attB* recombination (substrate pPB) reached 80% at 200–400 nM integrase, and decreased slightly at higher integrase concentrations. For maximal recombination, an excess of integrase (or integrase and RDF) over substrate binding sites was required (in these experiments, the plasmid substrate concentration was 10 nM, so the concentration of integrase monomer-binding sites is 40 nM; two in each *att* site). Excess integrase might be required because non-specific binding to DNA reduces the amount of integrase available to bind at the *att* sites. There was no recombination at all between *attL* and *attR* (substrate pLR) after 3 h of incubation with integrase in the absence of RDF, in agreement with existing *in vitro* data (19). These results illustrate the key ‘unidirectionality’ property of integrase-catalysed recombination; *attP* × *attB* recombination is highly efficient in the presence of integrase alone, while *attL* × *attR* recombination is not detectable. Furthermore, since different proportions of pLR and pPB were produced depending on whether pPB or pLR was used as the initial substrate (in the absence of RDF), one or both of these reactions must not be at equilibrium after 3 h. The possible reasons for this disequilibrium will be discussed below.

The presence of RDF enables recombination of the pLR substrate (Figure 2C and 4B) and inhibits recombination of the pPB substrate (Figures 2B and 4A), in agreement with previous observations (12). Recombination of the pLR substrate (giving pPB) was 70% after 3 h (200 nM integrase and 800 nM RDF; Figure 4B), whereas recombination of pPB (giving pLR) under the same conditions was only 12% (Figure 4A); again, one or both of these reactions has not reached equilibrium.

We then measured the kinetics of recombination in time course reactions with 400 nM ϕC31 integrase and 10 nM of plasmid substrate, in the absence (for pPB reactions) or presence (for pLR reactions) of 800 nM of RDF. In both cases, the recombinant product level quickly reached a maximum (Figure 4E), but not all of the substrate was recombined, in agreement with our 3-h data (Figure 2B and C) and published results (11). A possible explanation for incomplete recombination was that integrase loses activity over the course of the experiments. We therefore carried out experiments to determine the persistence of integrase activity when pre-incubated under our reaction conditions in the absence or presence of DNA and RDF (Supplementary Figure S5). We observed slow loss of integrase activity when incubated over 3 h on its own, but the presence of plasmid DNA or RDF substantially reduced this loss of activity. Substrate plasmid DNA had the strongest protective effect, such that integrase retained substantial activity for 3 h in the presence of substrate DNA. We conclude that incomplete recombination is not a consequence of integrase inactivation.

### Model development

#### Model 0. Sequence and energetics of reaction steps

Optimization of our models of integrase reactions proceeded through a number of iterations, as we assessed different reaction schemes and manually adjusted the parameters (constrained as described in ‘Materials and Methods’ section) to match the experimental data. The simplest version (Model 0; Figure 1C) captures the essential steps of the integrase-mediated reactions (14). We make the following assumptions. (i) All recombination reactions are intramolecular; that is, they occur between the two *att* sites in a single plasmid. Our experimental analysis confirms that this is a good approximation; the amount of plasmid dimer (the most abundant intermolecular product) is typically <5% of total product (data not shown). (ii) Integrase monomers interact to form dimers in solution, which then bind to the DNA. This assumption is supported by published data (12,19,23). (3) Integrase dimers have equal affinities for the two *att* sites in each substrate (e.g. *attP* and *attB* in pPB; *attL* and *attR* in pLR). The *attP* × *attB* reaction in the absence of RDF (P × B (−RDF)) thus starts with binding of two dimers of integrase to the *att* sites in the pPB plasmid substrate (step b1, forming PB–int; Figure 1C). The two integrase dimers then interact to form a tetramer, thereby bringing the two *att* sites together, in a step called synapsis (step s1, forming PB–int_s_). These two steps are modelled to be energetically favourable (Figure 3A), in agreement with experimental studies (12,19).

Recombination (strand exchange) then takes place (step r1, forming the LR product synapse LR–int_s1_). It is known that strand exchange is a complex process involving strand cleavages, subunit rotation and strand re-ligations, steps that are likely to be accompanied by protein conformational changes (1,24,25). However, for simplicity these are all condensed into a single step in our model. The current view of the strand exchange process for the serine integrases suggests that it is overall approximately isoenergetic, and thus its complexities will not affect directionality. We also note here that ∼50% of PB–int_s_ synapses are predicted to be incompetent for strand exchange because the two *att* sites are misaligned ‘in antiparallel’ (1,7,11,24,26). In these synapses, the two *att* sites must dissociate and reassociate to reach a strand exchange-proficient synapse. However, this factor is subsumed in the model into the strand exchange step r1, which will be slower than if all synapses were strand exchange-proficient. The LR synapse (LR–int_s1_) then dissociates (‘desynapsis’) to form the pLR product plasmid with two bound integrase dimers (step s2 (Model 0), forming LR–int). Desynapsis in step s2 is assumed in Model 0 to be favourable (Figure 3A), as has been proposed elsewhere (11; see ‘Discussion’ section). The final step (step b2) is dissociation of the integrase dimers from the LR product; however, the equilibrium should favour the integrase–LR complex (LR–int) at the integrase concentrations used here (19) and the product will remain protein-bound (Figure 3A).

Model 0 also describes the L × R reaction in the presence of RDF (L × R (+RDF)). The current structure-based hypothesis (14) proposes 1:1 stoichiometry of integrase to RDF. Our data support this by showing that RDF reaches its maximal effect, both in stimulation of the L × R reaction and inhibition of the P × B reaction, when its concentration equals or exceeds that of integrase (Figures 2 and 4). We therefore assume that each dimer of integrase binds to two monomers of RDF in solution (Supplementary Figure S2A) to form the productive int2–rdf2 complex. These complexes then bind to the two *att* sites in pLR, forming LR–int–rdf (step b3; Figure 1C). RDF might also bind to pre-formed integrase–DNA complexes, but preliminary simulations showed that inclusion of this process does not change the kinetics, so it was ignored for simplicity. The two DNA–integrase–RDF complexes then interact (synapsis) (step s3, forming LR–int–rdf_s_). Similarly to the P × B (−RDF) reaction, the binding of integrase–RDF complexes to pLR and formation of the synapse (LR–int–rdf_s_) are assumed to be energetically favourable steps (Figure 3B). Next, the LR synapse undergoes strand exchange (step r2, forming the PB product synapse PB–int–rdf_s1_). The synapse PB–int–rdf_s1_ then dissociates (‘desynapsis’; step s4 (Model 0), forming PB–int–rdf). Finally, dissociation of the two int2–rdf2 complexes from the recombinant *att* sites (step b4) would generate unbound pPB. However, as for the P × B (−RDF) reaction described above, protein-bound pPB is expected to be energetically favoured.

Model 0 also includes unproductive pLR complexes formed with two integrase dimers and one, two, or three RDF monomers (Supplementary Figure S2). The model assumes that only complexes containing four molecules of RDF are productive. The effects of insufficient RDF might be at pre- or post-synaptic steps. The formation of unproductive complexes reduces the amounts of reaction products in the L × R (+RDF) reaction when the RDF:integrase ratio is reduced below 1:1, in agreement with the data (Figures 2C and 4B). However, at RDF:integrase ratios greater than 1:1, integrase is present mainly in int2–rdf2 complexes with RDF. The L × R reaction thus proceeds towards the production of pPB product by forming LR–int–RDF complexes, and not competing (unproductive) LR–int complexes.

Our models ignore possible effects on the energetics of the reaction due to topological changes in the plasmid DNA during recombination. Current data suggest that the products of an inversion reaction of a supercoiled plasmid substrate are likely to comprise a complex mixture of topologies including a large proportion of knotted molecules, and it is also likely that these ‘knotting’ topological changes along with associated changes in DNA linking number are energetically favourable overall (1,18,27). However, the observed directionality of integrase-mediated recombination cannot be accounted for by any energetic bias as a result of these topological changes, because similar changes accompany both P × B and L × R reactions, and also would accompany secondary reactions of the recombinant products. In addition, we do not explicitly model any possible effects of the supercoiled structure of plasmid DNA on the affinity of integrase dimers for the recombination sites, or on the rate of binding. Finally, we simplified our analysis by ignoring the possibility of formation of unproductive ‘dead-end’ DNA–integrase complexes. Although in principle such complexes might affect reaction yields, their existence and abundance are unknown, and their formation cannot account for the directionality of the reactions.

#### Conservation of energy during integrase reactions. Limitations of Model 0

As noted above, the free energies of the substrate and recombinant product DNA molecules are expected to be about equal (ignoring possible changes in DNA topology; see above). The conservation of energy in a chain of reversible reactions in a closed cycle (and also between isoenergetic states) can be formally described by the so-called Wegscheider's condition (28), which requires that the product of all the equilibrium constants must be equal to one, or equivalently that the sum of free energy changes must be zero. Therefore, energetically favourable steps in our models must be balanced by unfavourable steps. In Model 0, all of the steps of the P × B (−RDF) reaction, from pPB substrate to integrase-bound LR–int product, are energetically favourable (Figure 3A), an overall negative change in free energy (ΔG) being necessary to drive the reaction towards predominantly recombinant product. This series of favourable steps is balanced by a single unfavourable step with positive ΔG, the dissociation of integrase dimers from LR–int to form free pLR (Figure 3A). In order to account for the favourable conversion of pPB to pLR whilst substrate and product DNA molecules have equal free energies, the binding energy (and thus affinity) of integrase for pLR must be modelled to be higher than that for pPB. However, this is in disagreement with experimentally determined affinities of integrase for *attP, attB, attL* and *attR* sites, which are all quite similar (19).

**Figure 5.**
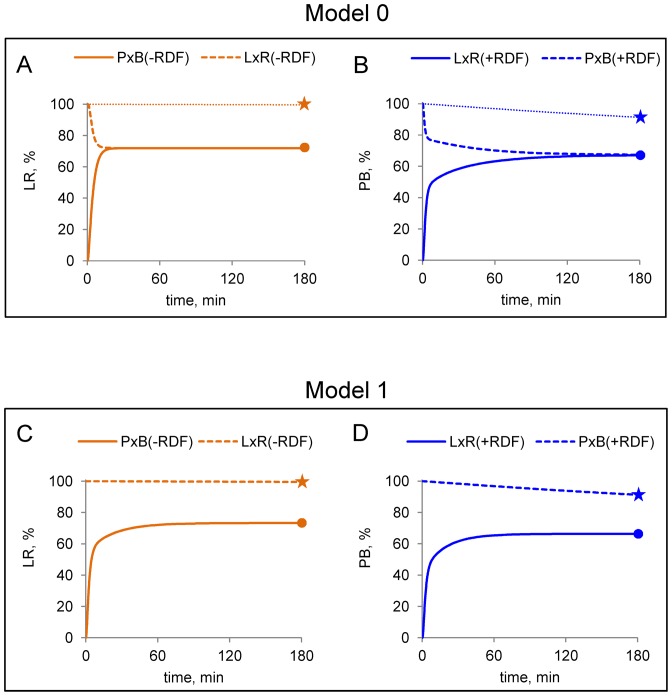
Modelled time courses of ϕC31 integrase-mediated recombination reactions. Reactions in the absence or presence of RDF are shown by orange and blue lines respectively, for Model 0 (**A** and **B**) or Model 1 (**C** and **D**). Simulations were performed at 400 nM integrase, 10 nM plasmid substrate (pPB or pLR) and 800 nM RDF (in parts B and D). For Model 0, parameters were fitted to the time course data from the ‘permitted’ reactions P × B (−RDF) and L × R (+RDF). For comparison, experimental data at 3-h time points are shown as symbols (P × B (−RDF), orange circles; L × R (−RDF), orange stars; L × R (+RDF), blue circles; P × B (+RDF), blue stars). In A and B, fine dotted lines show the experimentally observed time courses for the ‘forbidden’ (L × R (−RDF) and P × B (+RDF)) reactions.

A second problem is that in Model 0, all the reactions quickly approach equilibrium in both directions, yielding similar ratios of pPB to pLR in 3 h, regardless of whether the reaction starts from pPB or pLR, in contradiction to our experimental data (Figure 5A and B). The required difference in rates cannot be introduced simply by slowing down one of the reactions in the ‘forbidden’ direction. For instance, slowing down the L × R (−RDF) reaction by reducing the rate constant for the reverse of step s2 in Model 0 (formation of the LR–int_s1_ synapse from LR–int), would require an identical reduction of the rate constant for the same step in the forward (pPB to pLR) direction to keep the equilibrium constant for this step unchanged. These changes would reduce the rate at which the reaction approaches equilibrium in both directions, in disagreement with our data.

#### An improved model

**Figure 6.**
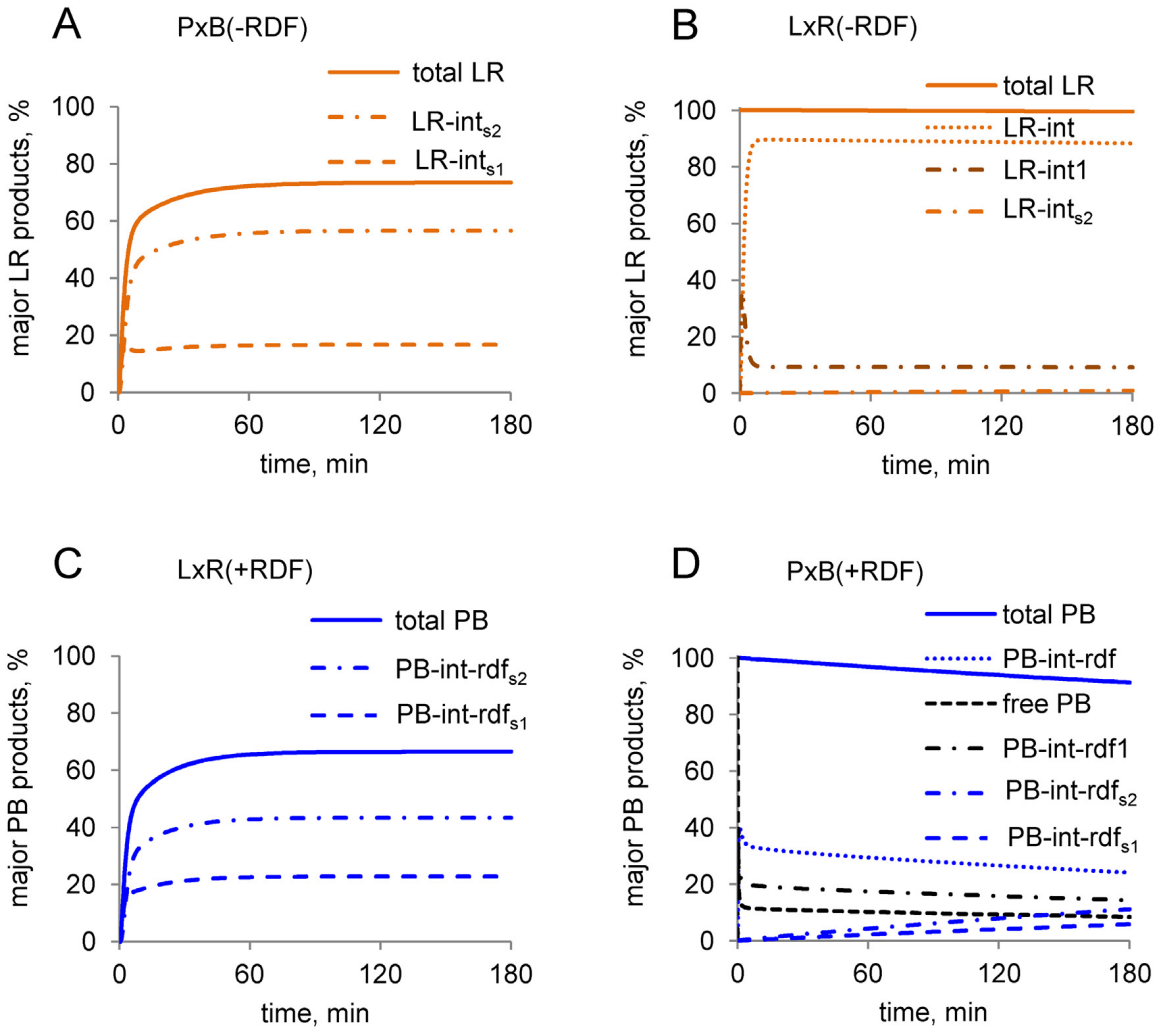
Simulated time courses of the amounts of abundant DNA-containing species in Model 1. (**A** and **B**) Reactions without RDF. Graphs show the amounts of the most abundant pLR species. These are LR synapses (LR–int_s1_, LR–int_s2_) in A and LR complexes with one (LR–int1) or two (LR–int) integrase dimers in B. (**C** and **D**) Reactions with RDF. Graphs show the amounts of the most abundant pPB species. These are PB synapses (PB–int–rdf_s1_, PB–int–rdf_s2_) in C and D, and free pPB, pPB bound by one (PB–int–rdf1) or two (PB–int–rdf) int2–rdf2 complexes in D. Note that the ‘permitted’ reactions (A and C) quickly approach equilibrium, with predominant formation of the final products LR–int_s2_ (A) or PB–int–rdf_s2_ (C), in contrast to the ‘non-permitted’ reactions (B and D). Simulations were for 400 nM integrase, 10 nM pPB or pLR substrate and 800 nM RDF (for C and D).

To resolve the problems in Model 0 discussed above, we hypothesized the existence of two additional synaptic complex species (LR–int_s2_ and PB–int–rdf_s2_; Figure 1C). This gave us Model 1, as used in most of our simulations. As described below, this modification allowed us to equalize the affinity of integrase for pPB and pLR, and to slow down the approach to equilibrium in the ‘forbidden’ L × R (−RDF) and P × B (+RDF) reactions. We propose that in the P × B (−RDF) reaction, a second synaptic complex LR–int_s2_ is formed from LR–int_s1_ (step mod, Figure 1C). LR–int_s2_ has lower free energy than LR–int_s1_ (Figure 3C) and is the most abundant product of the P × B (−RDF) reaction in Model 1 (Figure 6A). Structural studies have revealed a potential molecular basis for this modification step, a conformational change in the synapse ((14); see ‘Discussion’ section). The unfavourable dissociation of LR–int_s2_ to form unbound pLR can then occur in two stages in Model 1; desynapsis of the LR–int_s2_ synapse to form LR–int, followed by dissociation of integrase dimers from LR–int (Figure 3C). Splitting the dissociation of LR–int_s2_ into two energetically unfavourable steps, in contrast to the single highly unfavourable dissociation step in Model 0 (Figure 3A), allows the model to account for efficient conversion of pPB to pLR, while permitting integrase to have a similar affinity for all four types of recombination site. In Model 1, integrase binding to *attL* and *attR* sites is only twice as strong as binding to *attP* and *attB* (Supplementary Tables S1), in better agreement with published data (19).

Likewise, we hypothesized an additional synaptic complex PB–int–rdf_s2_ in the ‘reverse’ L × R (+RDF) reaction, with its unfavourable desynapsis in step s4 (Figures 1C and 3D).

To correct the problem that the ‘forbidden’ reactions (L × R (−RDF) and P × B (+RDF)) reach equilibrium too quickly in Model 0 (Figure 5A and B), we hypothesize that dissociation and formation (desynapsis/synapsis) of the stable synaptic complexes (LR–int_s2_ and PB–int–rdf_s2_) are slow (Figure 1C). For the P × B (−RDF) reaction, we therefore substantially decreased the rate constants of *s2* (both forward and reverse). This allowed us to account for the observed difference in pLR:pPB ratio at the end of reactions started from pPB or pLR (Figure 5C), as a consequence of very slow formation of LR–int_s2_ in the L × R (−RDF) reaction, and thus a very slow approach to equilibrium. On a short timescale, the L × R (−RDF) reaction proceeds only as far as binding of the pLR substrate by integrase dimers in Model 1, as discussed further below. Similarly, for the L × R (+RDF) reaction, we hypothesize that the forward and reverse rate constants of step s4 (desynapsis/synapsis of the stable synaptic complex PB–int–rdf_s2_ to PB–int–rdf) are low. We can thus explain the observed difference in the pLR:pPB ratio at the end of reactions started from pLR or pPB in the presence of RDF, by the very slow approach of the P × B (+RDF) reaction to equilibrium (Figure 5D).

Finally, to account for the observed inhibition of reactions at high concentrations of integrase (Figure 4A), we hypothesize in Model 1 that integrase dimers from solution can associate with dimers already bound at single *att* sites, so that an *att* site may be bound by an integrase tetramer and thus become incompetent for synapsis (Supplementary Figure S2C) (29).

### Simulated kinetics of reactions with plasmid DNA substrates

Model 1 accurately matches our data for the kinetics of the P × B (−RDF) and L × R (+RDF) reactions on plasmid substrates (Figure 4E). The model also has an excellent fit to the experimentally determined product levels after 3 h recombination reactions at a wide range of integrase and RDF concentrations (compare Figure 4A and B with C and D).

Model 1 predicts that the P × B (−RDF) reaction rapidly approaches equilibrium, reaching 68% of pLR recombinant product in 30 min and 75% of pLR at final equilibrium, at an integrase concentration of 400 nM (Figure 5C). The LR–int_s1_ and LR–int_s2_ synaptic complexes represent the two major pLR fractions at equilibrium (Figures 3C and 6A). Model 1 predicts very slow kinetics for the L × R (−RDF) reaction (Figure 5C), because most of the pLR substrate is initially bound to integrase dimers in the non-productive LR–int complex (Figure 6B), which is kinetically stable and is converted only very slowly to the synaptic complex LR–int_s2_ (Figure 6B; Supplementary Figure S6B). As a result, less than 0.2% of pPB product is formed after 1 h (compared to 25% of pPB at equilibrium, which would take several days to approach). The integration reaction forming *attL* and *attR* from *attP* and *attB* in the absence of RDF (P × B (−RDF)) should thus be effectively irreversible under natural *in vivo* conditions, where events such as chromosome replication and cell division would typically occur on a faster timescale. Sequestration of the free LR product into a non-productive complex with integrase (LR–int) might be a viral strategy to avoid spontaneous provirus excision in the absence of RDF.

At high RDF concentrations, nearly all integrase is complexed with RDF; both P × B and L × R reactions are thus channelled into the RDF-dependent branch of the pathway (blue arrows in Figure 1C). The L × R (+RDF) reaction approaches equilibrium rapidly in Model 1, reaching 62% pPB recombinant product after 30 min (compared to 67% pPB at equilibrium; Figures 5D and 6C) at 400 nM integrase and 800 nM RDF. The P × B (+RDF) reaction approaches equilibrium much more slowly, reaching only 3.2% pLR after 60 min (compared to 33% at eventual equilibrium) (Figure 5D), because the initially formed PB–int–rdf complex is converted only very slowly to synaptic complexes PB–int–rdf_s2_, which can then equilibrate with PB–int–rdf_s1_ and LR–int–rdf_s_ (Figures 3D and 6D; Supplementary Figure S6D). Therefore, the model explains the observed inhibition of the P × B reaction by RDF (Figure 4A and C; (12,13)); the pPB substrate is trapped in pre-synaptic integrase–RDF complexes.

### Kinetics of reactions with linear DNA substrates

**Figure 7.**
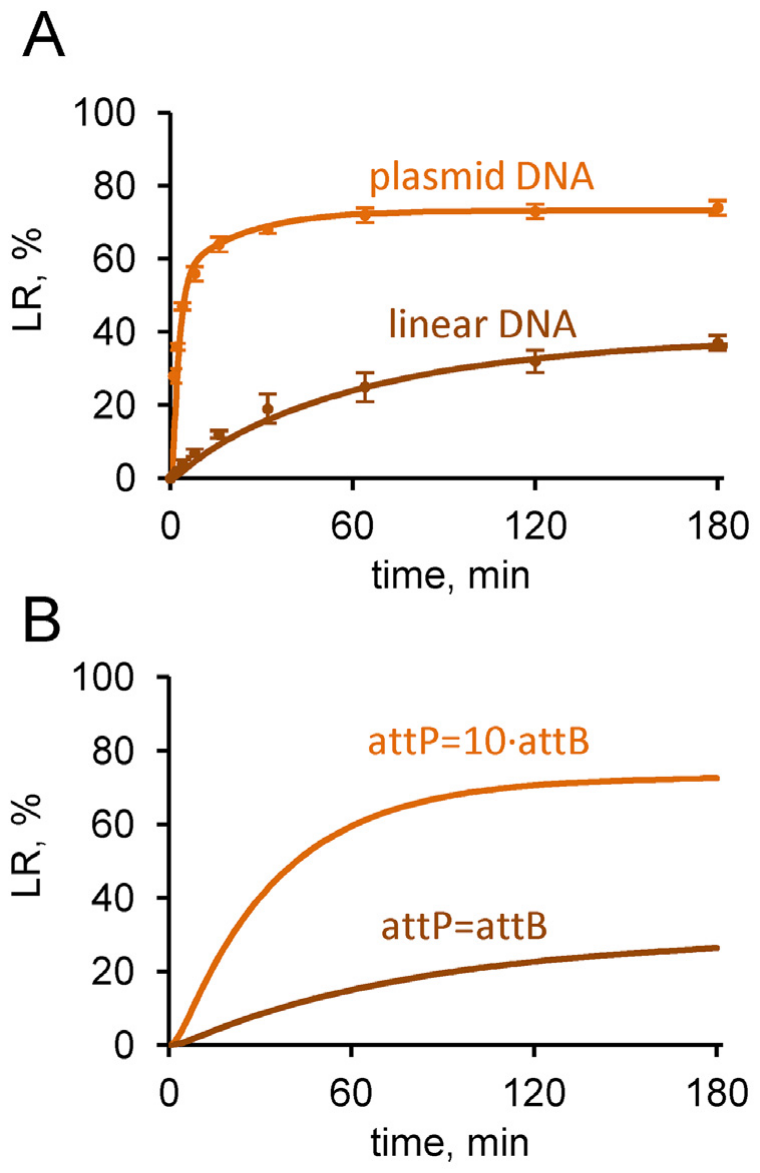
Kinetics of ϕC31 integrase-mediated recombination of linear *attP* and *attB* substrates. (**A**) Comparison of experimentally measured and modelled time courses with plasmid and linear substrates. Experimental data are shown by symbols and simulations by lines. Experiments and simulations were done with 400 nM integrase and 10 nM pPB, or 10 nM each of *attP* and *attB*. Plasmid data are the same as in Figure 4E. (**B**) Simulated time courses with equal or different amounts of *attP* and *attB* substrates. Concentrations of substrates were 3 nM *attP* and 3 nM *attB* for the brown line, 3 nM *attB* and 30 nM *attP* for the orange line; integrase was at 200 nM. The product amount is shown relative to the total amount of *attB*.

In the above analysis, both P × B and L × R reactions were intramolecular (between two *att* sites within a single supercoiled plasmid molecule), whereas the natural P × B reaction is intermolecular (between sites on the phage and bacterial genomic DNA), and intermolecular reactions (both P × B and L × R) are required for many proposed applications. Therefore we measured the experimental kinetics of the P × B (−RDF) reaction with linear DNA substrates. Recombination between linear (oligonucleotide) *attP* and *attB* substrates is slower than intramolecular recombination of a plasmid substrate (Figure 7A). We developed a modified version of Model 1 for linear substrates (Supplementary Figure S1 and Supplementary Text S1.2.2). Intramolecular synapsis in a supercoiled plasmid substrate is expected to be more favourable than intermolecular synapsis between sites on linear molecules (27,30). Supercoiling is also expected to increase the rate of the reaction (step r1), as it can be coupled to energetically favourable loss of supercoils and might also affect the stability of integrase complexes with *att* sites (18,27,30,31). Therefore we assumed in our modified Model 1 for linear substrates that synapsis is bimolecular and the recombination steps are slower compared to the model for plasmid substrates. These changes resulted in a lower rate of the P × B (−RDF) reaction in our simulations (10 nM *attP* and 10 nM *attB*) compared to 10 nM plasmid substrate under the same conditions (Figure 7A). The level of *attL* and *attR* products reaches 40% over 3 h in our simulations, in agreement with the data. The model predicts that conversion of one substrate (e.g. *attB*) to product can be enhanced by increasing concentration of the second substrate (e.g. *attP;* Figure 7B), as expected and as observed experimentally (11,22).

## DISCUSSION

Our optimized model (Model 1) reproduces the observed behaviour of the ϕC31 site-specific recombination system and provides a putative molecular explanation for directionality and the action of RDF.

Two previous works have also modelled the kinetics of recombination by serine integrases (8,32), but both studies make mechanistic assumptions that do not accord with experimental observations. Both previous models assume that RDF binds only to integrase that is pre-bound to DNA, while we allow RDF to bind to integrase that is in solution. Binding of RDF to integrase in the absence of DNA has been demonstrated experimentally for ϕC31 integrase (12), and incorporating this binding in our model allows recombination to respond to the RDF:integrase stoichiometry in a way that corresponds to our experimental observations. Both previous models account for the directionality of recombination by assuming that the recombination steps (conversion of PB to LR in a complex with integrase alone, and of LR to PB in an integrase–RDF complex) are strictly irreversible, whereas we assume more realistically that all reaction steps are (in principle) reversible. Bowyer *et al*. suggest that the observed incomplete conversion of substrate to product *in vitro* is due to irreversible inactivation of integrase during the course of the reaction (32). We present experimental data demonstrating that integrase retains substantial activity for the full length of our reactions (Supplementary Figure S5), and therefore do not incorporate integrase inactivation in our modelling. In addition, both previous models lack energetic constraints on the cyclic conversion of PB to LR and back to PB again, whereas in our model, cyclic reactions are constrained by Wegscheider's condition so that they do not violate the conservation of energy.

Our modelling is informed by the landmark structural analysis of Rutherford *et al*. (14–16), who have used crystal structures of a related serine integrase bound to its recombination site DNA to establish a structure-based hypothesis for the mechanism of directionality in recombination, in which a mobile coiled-coil (CC) domain plays a key role. It is proposed that during the P × B reaction, the substrate synapse is stabilized by interactions of CC domains that connect two different *att* sites (*attP* and *attB*) together (Figure 1B). After the strand exchange steps, dissociation of the LR product synapse is stimulated by switching of the CC domains to interactions within the integrase dimers bound to each single *att* site (Figure 1B, (14)). These interactions then block synapsis for the ‘forbidden’ L × R reaction. At a structural level, it is proposed that interactions of CC domains on a single *att* site are disfavoured in dimers bound to the (longer) *attP* and (shorter) *attB* sites, but are favourable in dimers bound to the intermediate-length *attL* and *attR* sites (1,14–16).

Our preliminary Model 0 conforms to the hypothesis of van Duyne *et al*. (14), in that the LR product synapse (LR–int_s1_) dissociates to give the pLR product plasmid bound by two integrase dimers (LR–int, Figure 1C) which is the final stable product of the P × B reaction. However, to drive equilibrium towards the LR product, Model 0 requires much higher affinity of integrase dimers for *attL* and *attR* sites than for *attP* or *attB* sites, whereas experimental data suggest similar affinities (19,23). To resolve this paradox, our final model (Model 1, Figure 1C) includes a new stable synaptic complex LR–int_s2_, which we propose to be the normal end-point of the P × B reaction. We hypothesize that LR–int_s1_, the immediate product of the strand exchange step, might convert to LR–int_s2_ by switching of the integrase CC domains from interactions between *attL* and *attR* sites to interactions on each single site, without dissociation of the synapse (Figure 1C; (1,14,15)). The LR–int_s2_ synapse is of lower free energy than the de-synapsed product LR–int (Figure 3C), allowing us to split the dissociation of the synapse over two steps (s2 and b2) and to use realistic binding affinities of integrase dimers for the *attL* and *attR* sites (19,23).

Stable integrase–product DNA synaptic complexes have not yet been observed experimentally in biochemical assays (22), though analogous species have been inferred in other recombinase systems (20). One possible explanation for this is that the product synaptic complexes do not survive the conditions used for gel electrophoresis. Another formal possibility is that the final kinetically stable end product of the P × B (−RDF) reaction that we refer to as LR–int_s2_ is not a synapse, but is pLR plasmid bound to two separate integrase dimers, in a conformation different from the LR–int complex formed after binding of integrase to pLR. The transition between these two configurations of LR–integrase complexes should be very slow accordingly to our model. The model and the existing data do not allow discrimination between these two possibilities.

We further hypothesize that interconversion of the synaptic complex LR–int_s2_ and the non-synapsed pLR plasmid bound by integrase dimers (LR–int) is very slow. This assumption allows Model 1 to account for the unidirectionality of P × B recombination in the absence of RDF (Figure 5D); the ‘reverse’ L × R reaction is very slow due to a large activation barrier for the formation of the LR–int_s2_ synapse from the integrase dimer-bound substrate LR–int, despite the favourable free energy change. A molecular explanation might be that the two CC domains of an integrase dimer bound to a single *attL* or *attR* site interact strongly in a way that blocks synapsis.

In the presence of RDF, integrase dimers bound to *attL* and *attR* must synapse readily, to initiate the L × R recombination reaction. As proposed by Rutherford *et al*. (14), RDF might interact with integrase and alter its conformation such that interactions of the CC domains within dimers bound to *attL* and *attR* are disfavoured, and thus synapsis (involving interactions of the CC domains between the two sites) is promoted. In our Model 1, this corresponds to rapid synapsis of *attL* and *attR* sites bound by integrase–RDF complexes (Figure 1C, step s3), in contrast to the very slow corresponding step (s2) in the absence of RDF. To account for the equilibrium in favour of product pPB plasmid, we again hypothesize a stable product synaptic complex (PB–int–rdf_s2_) which is the normal end-point of the reaction and slow interconversion of this synaptic complex with the product plasmid bound by two separate integrase dimer–RDF complexes (PB–int–rdf; step s4).

Conversion of substrates to recombinant products by ϕC31 integrase is never 100%, even at optimal concentrations of integrase and RDF (see ‘Results’ section). Our Model 1 accounts for this incomplete conversion as being primarily due to fast equilibration of synapsed forms of the substrate and product species (steps r1, mod of the P × B reaction, and steps r2, modr of the L × R reaction). The model therefore suggests that alterations to the equilibrium constants of these steps (by mutation of the proteins or *att* sites, or by changing the reaction conditions or substrate structure) could increase the conversion efficiency of the reactions, as might be desirable for many applications in biotechnology. For pPB recombination, a 2-fold increase of either of the equilibrium constants *K*_r1_ or *K*_mod_ (with a compensating change in *K*_s2_ to fulfil Wegscheider's condition) increases the pLR product yield at equilibrium from 76 to 86% (Supplementary Figure S3A and C). Likewise, a 2-fold increase of *K*_r2_ or *K*_modr_ for the pLR (+RDF) reaction increases the pPB product yield from 67 to 80%. Differences in the observed conversion efficiencies of natural serine integrases (33) might similarly be attributable to differences in the equilibrium constants of these sensitive steps.

## Supplementary Material

SUPPLEMENTARY DATA
