# Supplementary material for: The mechanism of ϕC31 integrase directionality: experimental analysis and computational modelling
Source: Nucleic Acids Res. 2016 Jul 7;44(15):7360–72. doi: 10.1093/nar/gkw616 (PMC5009753; doi:10.1093/nar/gkw616)
Supplement: SUPPLEMENTARY DATA [file supp_44_15_7360__index.html]

The mechanism of ϕC31 integrase directionality: experimental analysis and computational modelling — SUPPLEMENTARY DATA 

# The mechanism of ϕC31 integrase directionality: experimental analysis and computational modelling

## SUPPLEMENTARY DATA

- SUPPLEMENTARY DATA
