# Supplementary material for: The mechanism of ϕC31 integrase directionality: experimental analysis and computational modelling
Source: Nucleic Acids Res. 2016 Jul 7;44(15):7360–72. doi: 10.1093/nar/gkw616 (PMC5009753; doi:10.1093/nar/gkw616)
Supplement: SUPPLEMENTARY DATA [file supp_gkw616_nar-00642-z-2016-File009.pdf]

Supplementary Materials

The mechanism of ϕC31 integrase directionality: Experimental analysis and computational modelling

Alexandra Pokhilko, Jia Zhao, Oliver Ebenhöh, Margaret C. M. Smith, W. Marshall Stark and Sean D. Colloms

Table of Contents

S1.1 Experimental methods p. 2

S1.1.1 Oligonucleotide sequences p. 2

S1.1.2 Experiments to test the stability of integrase p. 3

S1.2 Modelling p. 4

S1.2.1 Model 1 description p. 4

S1.2.2 Model 1, modified for linear DNA substrates p. 9

S1.2.3 Model 0 description p. 12

S1.3 References p. 14

S1.4 Matlab code of Model 1 for plasmid substrates p. 15

S1.5 Supplementary figures p. 19

S1.6 Supplementary Tables 1-4 p. 27

S1.1 Experimental methods

S1.1.1 Oligonucleotide sequences

Experiments with linear DNA fragments used oligonucleotides with the sequences shown below, annealed to make 79 bp FAM-labelled *attP* and 86 bp Cy5-labelled *attB* double-stranded DNA fragments. The *att* sites are shown underlined with the central dinucleotides in bold.

***attP*-top:**
5'-AAAAAAAAAAAACACGGGGAAGCTTGAGCTCAGTAGTGCCCCAACT
GGGGTAACCT**TT**GAGTTCTCTCAGTTGGGGGCGTAGGGGAATTCT -3'

5'-FAM-*attP*-bot:
FAM 5'-AGAATTCCCCTACGCCCCCAACTGAGAGAACTC**AA**AGGTTACC
CCAGTTGGGGCACTACTGAGCTCAAGCTTCCCCGTG -3'

***attB*-top:**
5'-TGGCCGCGGTGCGGGTGCCAGGGCGTGCCC**TT**GGGCTCCCCGGGCGC
GTACTCCACCTCTGCAGGGATCCAAGTCCAGTGGACTT -3'

5'-Cy5-*attB*-bot:
Cy5 5'-AAGTCCACTGGACTTGGATCCCTGCAGAGGTGGAGTACGCGCCCG
GGGAGCCC**AA**GGGCACGCCCTGGCACCCGCACCGCGGCCA -3'

Plasmid substrates containing single *att* sites were constructed using the oligonucleotides shown below. See section S.1.1.2 for further details.

MCS-top
5'- CCGGCGATATCCCATGGGGATCCGAATTCGCATGCGAGCTCAGATCTG
GTACCTTTAAAC -3'

MCS-bot
5'- TCGAGTTTAAAGGTACCAGATCTGAGCTCGCATGCGAATTCGGATCC
CCATGGGATATCG -3'

***attP*-ES-top**
5'- CAGTAGTGCCCCAACTGGGGTAACCT**TT**GAGTTCTCTCAGTTGGGGGC
GTAGGGG -3'

***attP*-ES-bot**
5'- AATTCCCCTACGCCCCCAACTGAGAGAACTC**AA**AGGTTACCCCAGTTG
GGGCACTACTGAGCT -3'

***attB*-rev-ES-top**
5'- CAGGTGGAGTACGCGCCCGGGGAGCCC**AA**GGGCACGCCCTGGCACCC
GCACCGCGGG -3'

***attB*-rev-ES-bot**
5'- AATTCCCGCGGTGCGGGTGCCAGGGCGTGCCC**TT**GGGCTCCCCGGGC GCGTACTCCACCTGAGCT -3'

S1.1.2 Experiments to test the stability of integrase

To test the stability of integrase in recombination reaction buffer, proteins (4 μM integrase, or 4 μM integrase plus 8 μM RDF, in protein dilution buffer) were diluted 10-fold into reaction buffer and pre-incubated for up to 3 hours without DNA (Fig. S5A,B). Reaction volumes were typically 10 μl per time-point, and the final buffer composition for pre-incubation reactions was 27.5 mM Tris·HCl pH 7.5, 2.5 mM spermidine, 125 mM NaCl, 100 μM DTT, 5% glycerol, and 50 μg/ml BSA. Mixtures were incubated at 30 °C for different times before taking 9 μl aliquots and adding 1 μl of pPB or pLR substrate plasmid (final DNA concentration 10 nM). Mixtures were incubated for a further 3 hours at 30 °C to allow recombination, and then heated to 80 °C for 10 minutes to stop recombination. Reactions were analysed by restriction enzyme digestion with EcoRI and KpnI, followed by agarose gel electrophoresis, as described in the main text.

Integrase, or integrase plus RDF, were also pre-incubated for different lengths of time with plasmid DNA (10 nM) containing no (p0), or just one (pP or pB) recombination site (Fig. S5D). Proteins were diluted 1/10 as above, but into recombination buffer containing 10 nM plasmid DNA, and then incubated at 30 °C for different times. After pre-incubation, intermolecular recombination was initiated by adding 10 nM plasmid DNA containing the other *att* site (pP or pB), or for reactions containing p0 or no DNA, by adding 10 nM each of pP and pB. Recombination was allowed to proceed for a further 3 hours at 30 °C. Reactions were then stopped by heating to 80 °C, and analysed by restriction enzyme digestion with EcoRI followed by agarose gel electrophoresis.

Plasmid p0 (2476 bp) was constructed by ligating annealed MCS-top and MCS-bot oligonucleotides between the NgoMIV and XhoI sites of pMTL23 (1). Plasmid pP was then constructed by ligating annealed *attP*-ES-top and *attP*-ES-bot oligonucleotides between EcoRI and SacI sites of p0, while pB was constructed in an identical fashion using *attB*-rev-ES-top and *attB*-rev-ES-bot oligonucleotides. Intermolecular recombination between pP (2523 bp) and pB (2525 bp) yields a 5048 bp product that is cleaved by EcoRI to give fragments of 4987 bp and 61 bp (Fig. S5C).

S1.2 Modelling

S1.2.1 Model 1 description

The kinetics of reactions of ϕC31 integrase and its RDF gp3 (Fig. 1C) are described with the following system of ordinary differential equations (ODEs):

$$\begin{aligned}\frac{d[\text{LR}]}{dt} &= k_{+b2}\cdot[\text{LR - int2}] - k_{-b2}\cdot[\text{LR}]\cdot[\text{int2}] + k_{-b3}\cdot([\text{LR - int2 - rdf}] + \\ &[\text{LR - int2 - rdf2}]) - k_{+}\cdot[\text{LR}]\cdot([\text{int2 - rdf}] + [\text{int2 - rdf2}])\end{aligned} \qquad (1)$$

$$\frac{d[\text{int}]}{dt} = k_{-ii}\cdot(2\cdot[\text{int2}] + [\text{int2 - rdf}]) + k_{-ir}\cdot[\text{int - rdf}] - k_{+}\cdot(2\cdot[\text{int}]^2 + [\text{int}]\cdot[\text{int - rdf}] + [\text{int}]\cdot[\text{rdf}]) \qquad (2)$$

$$\frac{d[\text{int - rdf}]}{dt} = k_{+}\cdot([\text{int}]\cdot[\text{rdf}] - [\text{int}]\cdot[\text{int - rdf}] - 2\cdot[\text{int - rdf}]^2) - k_{-ir}\cdot[\text{int - rdf}] + k_{-ii}\cdot([\text{int2 - rdf}] + 2\cdot[\text{int2 - rdf2}]) \qquad (3)$$

$$\begin{aligned}\frac{d[\text{int2}]}{dt} &= k_{+}\cdot[\text{int}]^2 - k_{-ii}\cdot[\text{int2}] - k_{+}\cdot[\text{int2}]\cdot([\text{rdf}] + [\text{PB}] + [\text{PB - int2}]) - k_{-b2}\cdot[\text{int2}]\cdot([\text{LR}] + [\text{LR - int2}]) - \\ &k_{+s0}\cdot[\text{int2}]\cdot([\text{LR - int2 - rdf}] + [\text{LR - int2 - rdf2}] + [\text{PB - int2 - rdf}] + [\text{PB - int2 - rdf2}]) + k_{-ir}\cdot[\text{int2 - rdf}] + \\ &k_{-b1}\cdot([\text{PB - int2}] + [\text{PB - int4}]) + k_{-s01}\cdot([\text{LR - int4 - rdf}] + [\text{LR - int4 - rdf2}]) + k_{-s02}\cdot([\text{PB - int4 - rdf}] + [\text{PB - int4 - rdf2}]) + \\ &k_{+b2}\cdot([\text{LR - int2}] + [\text{LR - int4}]) - k_{+i}\cdot[\text{int2}]\cdot([\text{PB - int4}] + [\text{PB - int4 - rdf}] + [\text{PB - int4 - rdf2}] + [\text{PB - int4 - rdf3}] + \\ &[\text{PB - int4 - rdf4}]) + k_{-i}\cdot([\text{PB - int6}_{\text{i}}] + [\text{PB - int6 - rdf}_{\text{i}}] + [\text{PB - int6 - rdf2}_{\text{i}}] + [\text{PB - int6 - rdf3}_{\text{i}}] + [\text{PB - int6 - rdf4}_{\text{i}}])\end{aligned} \qquad (4)$$

$$\begin{aligned}\frac{d[\text{int2 - rdf}]}{dt} &= k_{+}\cdot([\text{int2}]\cdot[\text{rdf}] + [\text{int}]\cdot[\text{int - rdf}]) - (k_{-ir} + k_{-ii})\cdot[\text{int2 - rdf}] - k_{+}\cdot[\text{int2 - rdf}]\cdot[\text{rdf}] + k_{-ir}\cdot[\text{int2 - rdf2}] - \\ &k_{+}\cdot[\text{int2 - rdf}]\cdot([\text{PB}] + [\text{LR}]) - k_{+s0}\cdot[\text{int2 - rdf}]\cdot([\text{LR - int2}] + [\text{LR - int2 - rdf}] + [\text{LR - int2 - rdf2}] + [\text{PB - int2}] + \\ &[\text{PB - int2 - rdf}] + [\text{PB - int2 - rdf2}]) + k_{+b4}\cdot[\text{PB - int2 - rdf}] + k_{-s01}\cdot([\text{LR - int4 - rdf}] + [\text{LR - int4 - rdf2}] + \\ &[\text{LR - int4 - rdf3}]) + k_{-s02}\cdot([\text{PB - int4 - rdf}] + [\text{PB - int4 - rdf2}] + [\text{PB - int4 - rdf3}]) + k_{-b3}\cdot[\text{LR - int2 - rdf}]\end{aligned} \qquad (5)$$

$$\begin{aligned}\frac{d[\text{int2 - rdf2}]}{dt} &= k_{+}\cdot([\text{int2 - rdf}]\cdot[\text{rdf}] + [\text{int - rdf}]^2) - (k_{-ir} + k_{-ii})\cdot[\text{int2 - rdf2}] - k_{+}\cdot[\text{int2 - rdf2}]\cdot([\text{PB}] + [\text{LR}] + \\ &[\text{PB - int2 - rdf2}] + [\text{LR - int2 - rdf2}]) - k_{+s01}\cdot[\text{int2 - rdf2}]\cdot([\text{LR - int2}] + [\text{LR - int2 - rdf}] + ([\text{PB - int2}] + \\ &[\text{PB - int2 - rdf}]) + k_{+bi4}\cdot([\text{PB - int2 - rdf2}] + [\text{PB - int4 - rdf4}]) + k_{-s01}\cdot([\text{LR - int4 - rdf2}] + \\ &[\text{LR - int4 - rdf3}]) + k_{-s02}\cdot([\text{PB - int4 - rdf2}] + [\text{PB - int4 - rdf3}]) + k_{-bi3}\cdot([\text{LR - int2 - rdf2}] + [\text{LR - int4 - rdf4}])\end{aligned} \qquad (6)$$

$$\begin{aligned}\frac{d[\text{rdf}]}{dt} &= k_{-ir}\cdot([\text{int - rdf}] + [\text{int2 - rdf}] + [\text{int2 - rdf2}] + [\text{BP - int2 - rdf}] + [\text{LR - int2 - rdf}] + [\text{PB - int2 - rdf2}] + \\ &[\text{LR - int2 - rdf2}]) - k_{+}\cdot[\text{rdf}]\cdot([\text{int}] + [\text{int2}] + [\text{int2 - rdf}] + [\text{PB - int2}] + [\text{LR - int2}] + [\text{PB - int2 - rdf}] + [\text{LR - int2 - rdf}])\end{aligned} \qquad (7)$$

$$\begin{aligned}\frac{d[\text{PB - int2}]}{dt} &= k_{+}\cdot[\text{int2}]\cdot[\text{PB}] - k_{-b1}\cdot[\text{PB - int2}] - k_{+}\cdot[\text{PB - int2}]\cdot[\text{int2}] - k_{+s0}\cdot[\text{PB - int2}]\cdot \\ &([\text{int2 - rdf}] + [\text{int2 - rdf2}]) + k_{-b1}\cdot[\text{PB - int4}] + k_{-s02}\cdot([\text{PB - int4 - rdf}] + [\text{PB - int4 - rdf2}]) - \\ &k_{+}\cdot[\text{rdf}]\cdot[\text{PB - int2}] - k_{-ir}\cdot[\text{PB - int2 - rdf}]\end{aligned} \qquad (8)$$

$$\begin{aligned}\frac{d[\text{LR - int2}]}{dt} &= k_{-b2}\cdot[\text{int2}]\cdot([\text{LR}] - [\text{LR - int2}]) - k_{+b2}\cdot([\text{LR - int2}] - [\text{LR - int4}]) - k_{+s0}\cdot[\text{LR - int2}]\cdot([\text{int2 - rdf}] \\ &+ [\text{int2 - rdf2}]) + k_{-s01}\cdot([\text{LR - int4 - rdf}] + [\text{LR - int4 - rdf2}]) - k_{+}\cdot[\text{rdf}]\cdot[\text{LR - int2}] + k_{+ir}\cdot[\text{LR - int2 - rdf}]\end{aligned} \qquad (9)$$

$$\begin{aligned}\frac{d[\text{PB - int4}]}{dt} &= k_{+}\cdot[\text{int2}]\cdot[\text{PB - int2}] - k_{-b1}\cdot[\text{PB - int4}] - k_{+s}\cdot[\text{PB - int4}] + k_{-s1}\cdot[\text{PB - int}_{\text{s}}] - \\ &k_{+i}\cdot[\text{int2}]\cdot[\text{PB - int4}] + k_{-i}\cdot[\text{PB - int6}_{\text{i}}]\end{aligned} \qquad (10)$$

$$\frac{d[\text{LR - int4}]}{dt} = k_{-b2}\cdot[\text{int2}]\cdot[\text{LR - int2}] - k_{+b2}\cdot[\text{LR - int4}] - k_{-s2}\cdot[\text{LR - int4}] + k_{+s2}\cdot[\text{LR - int}_{\text{s2}}] \qquad (11)$$

$$\frac{d[\mathrm{LR\text{-}int_{s2}}]}{dt} = k_{-s2}\cdot[\mathrm{LR\text{-}int4}] - k_{+s2}\cdot[\mathrm{LR\text{-}int_{s2}}] + k_{+\mathrm{mod}}\cdot[\mathrm{LR\text{-}int_{s1}}] - k_{-\mathrm{mod}}\cdot[\mathrm{LR\text{-}int_{s2}}] \tag{12}$$

$$\frac{d[\mathrm{PB\text{-}int_{s}}]}{dt} = k_{+s}\cdot[\mathrm{PB\text{-}int4}] - k_{-s1}\cdot[\mathrm{PB\text{-}int_{s}}] - k_{+r}\cdot[\mathrm{PB\text{-}int_{s}}] + k_{-r}\cdot[\mathrm{LR\text{-}int_{s1}}] \tag{13}$$

$$\frac{d[\mathrm{LR\text{-}int_{s1}}]}{dt} = k_{+r}\cdot[\mathrm{BP\text{-}int_{s}}] - k_{-r}\cdot[\mathrm{LR\text{-}int_{s1}}] - k_{+\mathrm{mod}}\cdot[\mathrm{LR\text{-}int_{s1}}] + k_{-\mathrm{mod}}\cdot[\mathrm{LR\text{-}int_{s2}}] \tag{14}$$

$$\begin{aligned}\frac{d[\mathrm{PB\text{-}int2\text{-}rdf}]}{dt} &= k_{+}\cdot[\mathrm{int2\text{-}rdf}]\cdot[\mathrm{PB}] - k_{+b4}\cdot[\mathrm{PB\text{-}int2\text{-}rdf}] - k_{+s0}\cdot[\mathrm{PB\text{-}int2\text{-}rdf}]\cdot([\mathrm{int2}] + \\ &[\mathrm{int2\text{-}rdf}] + [\mathrm{int2\text{-}rdf2}]) + k_{-s02}\cdot([\mathrm{PB\text{-}int4\text{-}rdf}] + [\mathrm{PB\text{-}int4\text{-}rdf2}] + [\mathrm{PB\text{-}int4\text{-}rdf3}]) + \\ &k_{+}\cdot[\mathrm{rdf}]\cdot([\mathrm{PB\text{-}int2}] - [\mathrm{PB\text{-}int2\text{-}rdf}]) - k_{-ir}\cdot([\mathrm{PB\text{-}int2\text{-}rdf}] - [\mathrm{PB\text{-}int2\text{-}rdf2}])\end{aligned} \tag{15}$$

$$\begin{aligned}\frac{d[\mathrm{LR\text{-}int2\text{-}rdf}]}{dt} &= k_{+}\cdot[\mathrm{int2\text{-}rdf}]\cdot[\mathrm{LR}] - k_{-b3}\cdot[\mathrm{LR\text{-}int2\text{-}rdf}] - k_{+s0}\cdot[\mathrm{LR\text{-}int2\text{-}rdf}]\cdot([\mathrm{int2}] + \\ &[\mathrm{int2\text{-}rdf}] + [\mathrm{int2\text{-}rdf2}]) + k_{-s01}\cdot([\mathrm{LR\text{-}int4\text{-}rdf}] + [\mathrm{LR\text{-}int4\text{-}rdf2}] + [\mathrm{LR\text{-}int4\text{-}rdf3}]) + \\ &k_{+}\cdot[\mathrm{rdf}]\cdot([\mathrm{LR\text{-}int2}] - [\mathrm{LR\text{-}int2\text{-}rdf}]) - k_{-ir}\cdot([\mathrm{LR\text{-}int2\text{-}rdf}] - [\mathrm{LR\text{-}int2\text{-}rdf2}])\end{aligned} \tag{16}$$

$$\begin{aligned}\frac{d[\mathrm{PB\text{-}int2\text{-}rdf2}]}{dt} &= k_{+}\cdot[\mathrm{int2\text{-}rdf2}]\cdot[\mathrm{PB}] - k_{+b4}\cdot[\mathrm{PB\text{-}int2\text{-}rdf2}] + k_{+}\cdot[\mathrm{PB\text{-}int2\text{-}rdf}]\cdot[\mathrm{rdf}] - \\ &k_{+ir}\cdot[\mathrm{PB\text{-}int2\text{-}rdf2}] - k_{+}\cdot[\mathrm{PB\text{-}int2\text{-}rdf2}]\cdot[\mathrm{int2\text{-}rdf2}] + k_{+b4}\cdot[\mathrm{PB\text{-}int4\text{-}rdf4}] - k_{+s0}\cdot[\mathrm{PB\text{-}int2\text{-}rdf2}]\cdot \\ &([\mathrm{int2}] + [\mathrm{int2\text{-}rdf}]) + k_{-s02}\cdot([\mathrm{PB\text{-}int4\text{-}rdf2}] + [\mathrm{PB\text{-}int4\text{-}rdf3}])\end{aligned} \tag{17}$$

$$\begin{aligned}\frac{d[\mathrm{LR\text{-}int2\text{-}rdf2}]}{dt} &= k_{+}\cdot[\mathrm{int2\text{-}rdf2}]\cdot[\mathrm{LR}] - k_{-b3}\cdot[\mathrm{LR\text{-}int2\text{-}rdf2}] + k_{+}\cdot[\mathrm{LR\text{-}int2\text{-}rdf}]\cdot[\mathrm{rdf}] - \\ &k_{+ir}\cdot[\mathrm{LR\text{-}int2\text{-}rdf2}] - k_{+}\cdot[\mathrm{LR\text{-}int2\text{-}rdf2}]\cdot[\mathrm{int2\text{-}rdf2}] + k_{-b3}\cdot[\mathrm{LR\text{-}int4\text{-}rdf4}] - k_{+s0}\cdot[\mathrm{LR\text{-}int2\text{-}rdf2}]\cdot \\ &([\mathrm{int2}] + [\mathrm{int2\text{-}rdf}]) + k_{-s01}\cdot([\mathrm{LR\text{-}int4\text{-}rdf2}] + [\mathrm{LR\text{-}int4\text{-}rdf3}])\end{aligned} \tag{18}$$

$$\begin{aligned}\frac{d[\mathrm{PB\text{-}int4\text{-}rdf4}]}{dt} &= k_{+}\cdot[\mathrm{int2\text{-}rdf2}]\cdot[\mathrm{PB\text{-}int2\text{-}rdf2}] - k_{-b4}\cdot[\mathrm{PB\text{-}int4\text{-}rdf4}] - k_{-s4}\cdot[\mathrm{PB\text{-}int4\text{-}rdf4}] + \\ &k_{+s4}\cdot[\mathrm{PB\text{-}int\text{-}rdf_{s2}}] - k_{+i}\cdot[\mathrm{int2}]\cdot[\mathrm{PB\text{-}int4\text{-}rdf4}] + k_{-i}\cdot[\mathrm{PB\text{-}int6\text{-}rdf4_{i}}]\end{aligned} \tag{19}$$

$$\begin{aligned}\frac{d[\mathrm{LR\text{-}int4\text{-}rdf4}]}{dt} &= k_{+}\cdot[\mathrm{int2\text{-}rdf2}]\cdot[\mathrm{LR\text{-}int2\text{-}rdf2}] - k_{-b3}\cdot[\mathrm{LR\text{-}int4\text{-}rdf4}] - \\ &k_{+s}\cdot[\mathrm{LR\text{-}int4\text{-}rdf4}] + k_{-s3}\cdot[\mathrm{LR\text{-}int\text{-}rdf_{s}}]\end{aligned} \tag{20}$$

$$\begin{aligned}\frac{d[\mathrm{PB\text{-}int\text{-}rdf_{s2}}]}{dt} &= k_{+\mathrm{mod}}\cdot[\mathrm{PB\text{-}int\text{-}rdf_{s1}}] - k_{-\mathrm{mod}\,r}\cdot[\mathrm{PB\text{-}int\text{-}rdf_{s2}}] - \\ &k_{+s4}\cdot[\mathrm{PB\text{-}int\text{-}rdf_{s2}}] + k_{-s4}\cdot[\mathrm{PB\text{-}int4\text{-}rdf4}]\end{aligned} \tag{21}$$

$$\begin{aligned}\frac{d[\mathrm{PB\text{-}int\text{-}rdf_{s1}}]}{dt} &= k_{+r}\cdot[\mathrm{LR\text{-}int\text{-}rdf_{s}}] - k_{-r}\cdot[\mathrm{PB\text{-}int\text{-}rdf_{s1}}] + \\ &k_{-\mathrm{mod}\,r}\cdot[\mathrm{PB\text{-}int\text{-}rdf_{s2}}] - k_{+\mathrm{mod}}\cdot[\mathrm{PB\text{-}int\text{-}rdf_{s1}}]\end{aligned} \tag{22}$$

$$\begin{aligned}\frac{d[\mathrm{LR\text{-}int\text{-}rdf_{s}}]}{dt} &= k_{+s}\cdot[\mathrm{LR\text{-}int4\text{-}rdf4}] - k_{-s3}\cdot[\mathrm{LR\text{-}int\text{-}rdf_{s}}] - \\ &k_{+r}\cdot[\mathrm{LR\text{-}int\text{-}rdf_{s}}] + k_{-r}\cdot[\mathrm{PB\text{-}int\text{-}rdf_{s1}}]\end{aligned} \tag{23}$$

$$\begin{aligned}\frac{d[\mathrm{PB}]}{dt} &= k_{-b1}\cdot[\mathrm{PB\text{-}int2}] + k_{+b4}\cdot([\mathrm{PB\text{-}int2\text{-}rdf}] + [\mathrm{PB\text{-}int2\text{-}rdf2}]) - \\ &k_{+}\cdot[\mathrm{PB}]\cdot([\mathrm{int2}] + [\mathrm{int2\text{-}rdf}] + [\mathrm{int2\text{-}rdf2}])\end{aligned} \tag{24}$$

$$\begin{aligned}\frac{d[\mathrm{PB\text{-}int4\text{-}rdf}]}{dt} &= k_{+s0}\cdot([\mathrm{int2}]\cdot[\mathrm{PB\text{-}int2\text{-}rdf}] + [\mathrm{int2\text{-}rdf}]\cdot[\mathrm{PB\text{-}int2}]) - 2k_{-s02}\cdot[\mathrm{PB\text{-}int4\text{-}rdf}] - \\ &k_{+i}\cdot[\mathrm{int2}]\cdot[\mathrm{PB\text{-}int4\text{-}rdf}] + k_{-i}\cdot[\mathrm{PB\text{-}int6\text{-}rdf_{i}}]\end{aligned} \tag{25}$$

$$\frac{d[\mathrm{PB-int4-rdf2}]}{dt} = k_{+s0} \cdot ([\mathrm{int2}] \cdot [\mathrm{PB-int2-rdf2}] + [\mathrm{int2-rdf}] \cdot [\mathrm{PB-int2-rdf}] + [\mathrm{int2-rdf2}] \cdot [\mathrm{PB-int2}]) - 3k_{-s02} \cdot [\mathrm{PB-int4-rdf2}] - k_{+i} \cdot [\mathrm{int2}] \cdot [\mathrm{PB-int4-rdf2}] + k_{-i} \cdot [\mathrm{PB-int6-rdf2_i}] \quad (26)$$

$$\frac{d[\mathrm{PB-int4-rdf3}]}{dt} = k_{+s0} \cdot ([\mathrm{int2-rdf}] \cdot [\mathrm{PB-int2-rdf2}] + [\mathrm{int2-rdf2}] \cdot [\mathrm{PB-int2-rdf}]) - 2k_{-s02} \cdot [\mathrm{PB-int4-rdf3}] - k_{+i} \cdot [\mathrm{int2}] \cdot [\mathrm{PB-int4-rdf3}] + k_{-i} \cdot [\mathrm{PB-int6-rdf3_i}] \quad (27)$$

$$\frac{d[\mathrm{LR-int4-rdf}]}{dt} = k_{+s0} \cdot ([\mathrm{int2}] \cdot [\mathrm{LR-int2-rdf}] + [\mathrm{int2-rdf}] \cdot [\mathrm{LR-int2}]) - 2k_{-s01} \cdot [\mathrm{LR-int4-rdf}] \quad (28)$$

$$\frac{d[\mathrm{LR-int4-rdf2}]}{dt} = k_{+s0} \cdot ([\mathrm{int2}] \cdot [\mathrm{LR-int2-rdf2}] + [\mathrm{int2-rdf}] \cdot [\mathrm{LR-int2-rdf}] + [\mathrm{int2-rdf2}] \cdot [\mathrm{LR-int2}]) - 3k_{-s01} \cdot [\mathrm{LR-int4-rdf2}] \quad (29)$$

$$\frac{d[\mathrm{LR-int4-rdf3}]}{dt} = k_{+s0} \cdot ([\mathrm{int2-rdf}] \cdot [\mathrm{LR-int2-rdf2}] + [\mathrm{int2-rdf2}] \cdot [\mathrm{LR-int2-rdf}]) - 2k_{-s01} \cdot [\mathrm{LR-int4-rdf3}] \quad (30)$$

$$\frac{d[\mathrm{PB-int6_i}]}{dt} = k_{+i} \cdot [\mathrm{int2}] \cdot [\mathrm{PB-int4}] - k_{-i} \cdot [\mathrm{PB-int6_i}] \quad (31)$$

$$\frac{d[\mathrm{PB-int6-rdf4_i}]}{dt} = k_{+i} \cdot [\mathrm{int2}] \cdot [\mathrm{PB-int4-rdf4}] - k_{-i} \cdot [\mathrm{PB-int6-rdf4_i}] \quad (32)$$

$$\frac{d[\mathrm{PB-int6-rdf_i}]}{dt} = k_{+i} \cdot [\mathrm{int2}] \cdot [\mathrm{PB-int4-rdf}] - k_{-i} \cdot [\mathrm{PB-int6-rdf_i}] \quad (33)$$

$$\frac{d[\mathrm{PB-int6-rdf2_i}]}{dt} = k_{+i} \cdot [\mathrm{int2}] \cdot [\mathrm{PB-int4-rdf2}] - k_{-i} \cdot [\mathrm{PB-int6-rdf2_i}] \quad (34)$$

$$\frac{d[\mathrm{PB-int6-rdf3_i}]}{dt} = k_{+i} \cdot [\mathrm{int2}] \cdot [\mathrm{PB-int4-rdf3}] - k_{-i} \cdot [\mathrm{PB-int6-rdf3_i}] \quad (35)$$

The variables are defined as follows:

[int], [int2], [int2-rdf], [int2-rdf2] are the concentrations of the respective complexes of integrase and RDF in solution; [LR], [PB] are concentrations of free plasmid DNA (referred to as pLR and pPB respectively in the main text). Since both *attP* and *attB* (or *attL* and *attR*) sites are located on the same plasmid, a single variable (PB or LR) corresponds to the two sites (Fig. 1C).

[PB-int2], [LR-int2] are concentrations of the complexes of plasmid DNA with only one site occupied by an integrase dimer. [PB-int2-rdf], [PB-int2-rdf2], [LR-int2-rdf], and [LR-int2-rdf2] are concentrations of complexes containing an integrase dimer and one (-rdf) or two (-rdf2) RDF molecules. [PB-int4], [LR-int4], [PB-int4-rdf4], [LR-int4-rdf4] are the concentrations of DNA complexes consisting of two integrase dimers (not synapsed), with or without RDF (these complexes are shown as PB-int, LR-int, PB-int-rdf, LR-int-rdf respectively in Fig.1C). [PB-$\mathrm{int_s}$], [LR-$\mathrm{int_{s1}}$], [LR-$\mathrm{int_{s2}}$], [LR-int-$\mathrm{rdf_s}$], [PB-int-$\mathrm{rdf_{s1}}$], [PB-int-$\mathrm{rdf_{s2}}$] are the concentration of synapses (as shown in Fig. 1C).

[PB-int4-rdf], [PB-int4-rdf2], [PB-int4-rdf3], [LR-int4-rdf], [LR-int4-rdf2], [LR-int4-rdf3] are the concentrations of unproductive complexes of integrase and RDF with DNA (containing two dimers of integrase and one, two or three monomers of RDF), which do not participate in recombination. The reversible formation of these complexes (Fig. S2B) reduces reaction yield when RDF concentration is lower than integrase.

The observed inhibition of the P × B (-RDF) reaction by integrase concentrations higher than 200 nM (Fig. 4B) was described through the binding of additional integrase dimers to *att* sites which are already occupied by integrase dimers (hypothetically forming integrase tetramers by protein-protein interactions, preventing synapse formation with another *att* site already bound by an integrase dimer) (Fig. S2C). This results in the formation of non-productive complexes PB-int6$_i$, PB-int6-rdf$_i$, PB-int6-rdf2$_i$, PB-int6-rdf3$_i$, PB-int6-rdf4$_i$ at high integrase concentrations. We considered only inactivation of PB sites by excess integrase for simplicity (this was sufficient to describe the data) (Fig. 4, Fig.7). The unit of concentration is μM, and the unit of time is a minute. All simulations on plasmid DNA used a total DNA concentration of 10 nM, while integrase and RDF concentrations were varied as indicated in the figure legends.

The parameters k_{+n}, k_{-n} stand for the forward and reverse rate constants of step "*n*", with the forward direction from pPB towards pLR in the P × B (-RDF) reaction and from pLR towards pPB in the L × R (+RDF) reaction.

For each step, the equilibrium constant is defined as the ratio of forward to reverse rate constants: $K_{eq\_n}=k_{+n} / k_{-n}$. For DNA binding steps, it is convenient to consider dissociation constands K_d, where $K_d = K_{eq}$ for steps *b2* and *b4*, but 1/ K_{eq} for steps *b1* and *b3*. The equilibrium constants of recombination steps *r1* and *r2* are assumed to be equal ($K_{r1} = K_{r2} = K_r$).

The parameter values for all steps are shown in Table S1. This includes equilibrium constants for integrase (or integrase-RDF) binding to DNA (K_{b1}, K_{b3}) and dissociation from DNA (K_{b2}, K_{b4}); formation (K_{s1}, K_{s3}) and dissociation of synapses (K_{s2}, K_{s4}); recombination (K_r) and conformational changes in product synapses (K_{mod}, K_{modr}). Table S1 also includes dissociation constants of binding of two integrase molecules (with or without RDF) in solution (K_{ii}) (Fig. S2A); formation of integrase-RDF complexes (K_{ir}) (Fig. S2A); unproductive binding of integrase dimers to DNA at high integrase concentrations (K_i) (Fig. S2C); formation of unproductive tetrameric complexes of integrase with intermediate numbers (one, two, or three) of RDF molecules (K_{s01} for LR and K_{s02} for PB complexes) (Fig.

S2B). The rate constants for integrase (or integrase-RDF) binding to DNA were assumed to be equal for steps *b1, b3, b4,* while the rate constant of integrase binding to LR was reduced to account for the observed delay in the L × R (+RDF) reaction after premixture of the pLR substrate with integrase (2). The rate constants for formation of the product synapses (steps *s2* and *s4*) were assumed to be low, as described in the Results section. The values of all parameters were chosen to fit existing data and our new data (Fig. 4, Table S4). Most parameters are uniquely constrained by the observed system properties. In particular, the affinities of binding of integrase with or without RDF to DNA ($1/K_{b1}$, K_{b2}, $1/K_{b3}$, K_{b4}, see Table S1) are constrained by the observed dependence of reaction yields (the final % of reaction product) on integrase and RDF concentrations (Fig. 4B, C; Fig. S3E shows an effect of K_{b1} on the reaction yields as an example). The equilibrium constants for the formation and dissociation of synapses (K_{s1}, K_{s2}, K_{s3}, K_{s4}) and the rate constants for synapsis, recombination and unproductive integrase binding to DNA (k_{+s}, k_{+r}, k_{+s0}, k_{+i}) are constrained by the reaction kinetics (Fig. 4A, Fig. S3B). The equilibrium constants for modification of the product synapses (K_{mod}, K_{modr}) are strongly constrained by the observed yields of the products, as described in the Discussion. The equilibrium constants of the recombination steps (K_r) were chosen to be equal to 1, based on existing data demonstrating the easy reversibility of recombination in other serine recombinase systems (3). The rate constants for binding of integrase complexes to DNA and formation of protein complexes were chosen to be within typically observed ranges for the rates of recombinase binding to DNA and protein-protein interactions (4, 5). The rate constants for the slow steps *s2, s4* of product synapse dissociation were chosen to describe the observed slow accumulation of DNA products during the L × R (-RDF) and P × B (+RDF) reactions (Fig. 5C, D; Fig. 4B, C, Fig. S3C). The dissociation constants for unproductive integrase binding to DNA (K_i; Fig. S2C) were fitted to the kinetic data and data on inhibition of the P × B reaction at high integrase concentrations (Fig. 4B). The parameters for the formation of unproductive complexes of integrase tetramers with the substrate DNA, containing less than 4 molecules of RDF (PB-int4-rdf, PB-int4-rdf2, PB-int4-rdf3, LR-int4-rdf, LR-int4-rdf2, LR-int4-rdf3) (K_{s01}, K_{s02}; Fig. S2B), together with the dissociation constants of integrase dimers and complexes with RDF (K_{ii}, K_{ir}; Fig. S2A) were fitted to the data (Fig. 4B, C, Fig. S3F).

The conservation of energy during conversion of PB to LR and LR to PB constrains the parameter ranges as discussed in Results. In particular, conservation of energy requires that multiplication of all equilibrium constants (in the same direction) is equal to 1 for both -RDF

and +RDF reactions (Fig. 1). These conditions result in the following constraints on the range of possible parameter values:

$$K_{b1}^2 \cdot K_{s1} \cdot K_{r1} \cdot K_{\mathrm{mod}} \cdot K_{s2} \cdot K_{b2}^2 = 1 \;;\; K_{b3}^2 \cdot K_{s3} \cdot K_{r2} \cdot K_{\mathrm{mod}r} \cdot K_{s4} \cdot K_{b4}^2 = 1 \tag{36}$$

Here we assumed that unbound pPB and pLR have the same free energy as discussed in the main text. The squares for integrase binding terms are related to two sequential bindings of integrase dimers or int2-rdf2 complexes (see scheme above). For the simulations shown in Supplementary Fig. S3 and S4, where the equilibrium constants of the recombination or modification steps were increased, the equilibrium constants of de-synapsis (K_{s2} or K_{s4}) were correspondingly decreased, to conserve the total energy changes. The Matlab code of Model 1 is presented at the end of Supplementary Materials.

S1.2.2 Model 1, modified for linear DNA substrates

The reaction scheme is shown in Fig. S1. The main difference between the models with plasmid and linear substrates is that for the plasmid reactions, intramolecular synapse formation is described by first order kinetics (Fig. 1C), whereas for reactions with linear DNA substrates, each containing one *att* site, intermolecular synapse formation has second order kinetics (Fig. S1). The kinetics of the reaction scheme with linear substrates is described with the following system of ODEs:

$$\frac{d[\mathrm{L}]}{dt} = k_{+b2} \cdot [\mathrm{L-int2}] - k_{-b2} \cdot [\mathrm{L}] \cdot [\mathrm{int2}] + k_{-b3} \cdot ([\mathrm{L-int2-rdf}] + [\mathrm{L-int2-rdf2}]) - k_{+} \cdot [\mathrm{L}] \cdot ([\mathrm{int2-rdf}] + [\mathrm{int2-rdf2}]) \tag{1'}$$

$$\frac{d[\mathrm{R}]}{dt} = k_{+b2} \cdot [\mathrm{R-int2}] - k_{-b2} \cdot [\mathrm{R}] \cdot [\mathrm{int2}] + k_{-b3} \cdot ([\mathrm{R-int2-rdf}] + [\mathrm{R-int2-rdf2}]) - k_{+} \cdot [\mathrm{R}] \cdot ([\mathrm{int2-rdf}] + [\mathrm{int2-rdf2}]) \tag{2'}$$

$$\frac{d[\mathrm{int}]}{dt} = k_{-ii} \cdot (2 \cdot [\mathrm{int2}] + [\mathrm{int2-rdf}]) + k_{-ir} \cdot [\mathrm{int-rdf}] - k_{+} \cdot (2 \cdot [\mathrm{int}]^2 + [\mathrm{int}] \cdot [\mathrm{int-rdf}] + [\mathrm{int}] \cdot [\mathrm{rdf}]) \tag{3'}$$

$$\frac{d[\mathrm{int-rdf}]}{dt} = k_{+} \cdot ([\mathrm{int}] \cdot [\mathrm{rdf}] - [\mathrm{int}] \cdot [\mathrm{int-rdf}] - 2 \cdot [\mathrm{int-rdf}]^2) - k_{-ir} \cdot [\mathrm{int-rdf}] + k_{-ii} \cdot ([\mathrm{int2-rdf}] + 2 \cdot [\mathrm{int2-rdf2}]) \tag{4'}$$

$$\frac{d[\mathrm{int2}]}{dt} = k_{+} \cdot [\mathrm{int}]^2 - k_{-ii} \cdot [\mathrm{int2}] + k_{+b2} \cdot ([\mathrm{L-int2}] + [\mathrm{R-int2}]) - k_{-b2} \cdot [\mathrm{int2}] \cdot ([\mathrm{L}] + [\mathrm{R}]) - k_{+} \cdot [\mathrm{int2}] \cdot ([\mathrm{rdf}] + [\mathrm{B}] + [\mathrm{P}]) + k_{-ir} \cdot [\mathrm{int2-rdf}] + k_{-b1} \cdot ([\mathrm{B-int2}] + [\mathrm{P-int2}]) - k_{+i} \cdot [\mathrm{int2}] \cdot ([\mathrm{B-int2}] + [\mathrm{P-int2}]) + k_{-i} \cdot ([\mathrm{B-int4_i}] + [\mathrm{P-int4_i}]) \tag{5'}$$

$$\frac{d[\mathrm{int2-rdf}]}{dt} = k_{+} \cdot ([\mathrm{int2}] \cdot [\mathrm{rdf}] + [\mathrm{int}] \cdot [\mathrm{int-rdf}]) - (k_{-ir} + k_{-ii}) \cdot [\mathrm{int2-rdf}] - k_{+} \cdot [\mathrm{int2-rdf}] \cdot [\mathrm{rdf}] + k_{-ir} \cdot [\mathrm{int2-rdf2}] - k_{+} \cdot [\mathrm{int2-rdf}] \cdot ([\mathrm{B}] + [\mathrm{P}] + [\mathrm{L}] + [\mathrm{R}]) + k_{+b4} \cdot ([\mathrm{B-int2-rdf}] + [\mathrm{P-int2-rdf}]) + k_{-b3} \cdot ([\mathrm{L-int2-rdf}] + [\mathrm{R-int2-rdf}]) \tag{6'}$$

$$\frac{d[\mathrm{int2-rdf2}]}{dt} = k_{+} \cdot ([\mathrm{int2-rdf}] \cdot [\mathrm{rdf}] + [\mathrm{int-rdf}]^2) - (k_{-ir} + k_{-ii}) \cdot [\mathrm{int2-rdf2}] - k_{+} \cdot [\mathrm{int2-rdf2}] \cdot ([\mathrm{B}] + [\mathrm{P}] + [\mathrm{L}] + [\mathrm{R}]) + k_{+b4} \cdot ([\mathrm{B-int2-rdf2}] + [\mathrm{P-int2-rdf2}]) + k_{-b3} \cdot ([\mathrm{L-int2-rdf2}] + [\mathrm{R-int4-rdf4}]) \tag{7'}$$

$$\frac{d[\text{rdf}]}{dt} = k_{-ir}\cdot([\text{int-rdf}]+[\text{int2-rdf}]+[\text{int2-rdf2}]+[\text{B-int2-rdf}]+[\text{P-int2-rdf}]+[\text{L-int2-rdf}]+[\text{R-int2-rdf}]+[\text{B-int2-rdf2}]+[\text{P-int2-rdf2}]+[\text{L-int2-rdf2}]+[\text{R-int2-rdf2}]) - k_{+}\cdot[\text{rdf}]\cdot([\text{int}]+[\text{int2}]+[\text{int2-rdf}]+[\text{B-int2}]+[\text{P-int2}]+[\text{L-int2}]+[\text{R-int2}]+[\text{B-int2-rdf}]+[\text{P-int2-rdf}]+[\text{L-int2-rdf}]+[\text{R-int2-rdf}]) \tag{8'}$$

$$\frac{d[\text{B-int2}]}{dt} = k_{+}\cdot[\text{int2}]\cdot[\text{B}] - k_{-b1}\cdot[\text{B-int2}] - k_{+s}\cdot[\text{B-int2}]\cdot[\text{P-int2}] + k_{-s1}\cdot[\text{PB-int}_{\text{s}}] - k_{+}\cdot[\text{rdf}]\cdot[\text{B-int2}] + k_{-ir}\cdot[\text{B-int2-rdf}] - k_{+s0}\cdot[\text{B-int2}]\cdot([\text{P-int2-rdf}]+[\text{P-int2-rdf2}]) + k_{-s02}\cdot([\text{PB-int4-rdf}]+[\text{PB-int4-rdf2}]) - k_{+i}\cdot[\text{int2}]\cdot[\text{B-int2}] + k_{-i}\cdot[\text{B-int4}_{\text{i}}] \tag{9'}$$

$$\frac{d[\text{P-int2}]}{dt} = k_{+}\cdot[\text{int2}]\cdot[\text{P}] - k_{-b1}\cdot[\text{P-int2}] - k_{+s}\cdot[\text{B-int2}]\cdot[\text{P-int2}] + k_{-s1}\cdot[\text{PB-int}_{\text{s}}] - k_{+}\cdot[\text{rdf}]\cdot[\text{P-int2}] + k_{-ir}\cdot[\text{P-int2-rdf}] - k_{+s0}\cdot[\text{P-int2}]\cdot([\text{B-int2-rdf}]+[\text{B-int2-rdf2}]) + k_{-s02}\cdot([\text{PB-int4-rdf}]+[\text{PB-int4-rdf2}]) - k_{+i}\cdot[\text{int2}]\cdot[\text{P-int2}] + k_{-i}\cdot[\text{P-int4}_{\text{i}}] \tag{10'}$$

$$\frac{d[\text{L-int2}]}{dt} = k_{-b2}\cdot[\text{int2}]\cdot[\text{L}] - k_{+b2}\cdot[\text{L-int2}] - k_{+s2}\cdot[\text{L-int2}]\cdot[\text{R-int2}] + k_{-s2}\cdot[\text{LR-int}_{\text{s2}}] - k_{+}\cdot[\text{rdf}]\cdot[\text{L-int2}] + k_{-ir}\cdot[\text{L-int2-rdf}] - k_{+s0}\cdot[\text{L-int2}]\cdot([\text{R-int2-rdf}]+[\text{R-int2-rdf2}]) + k_{-s01}\cdot([\text{LR-int4-rdf}]+[\text{LR-int4-rdf2}]) \tag{11'}$$

$$\frac{d[\text{R-int2}]}{dt} = k_{-b2}\cdot[\text{int2}]\cdot[\text{R}] - k_{+b2}\cdot[\text{R-int2}] - k_{+s2}\cdot[\text{L-int2}]\cdot[\text{R-int2}] + k_{-s2}\cdot[\text{LR-int}_{\text{s2}}] - k_{+}\cdot[\text{rdf}]\cdot[\text{R-int2}] + k_{-ir}\cdot[\text{R-int2-rdf}] - k_{+s0}\cdot[\text{R-int2}]\cdot([\text{L-int2-rdf}]+[\text{L-int2-rdf2}]) + k_{-s01}\cdot([\text{LR-int4-rdf}]+[\text{LR-int4-rdf2}]) \tag{12'}$$

$$\frac{d[\text{PB-int}_{\text{s}}]}{dt} = k_{+s}\cdot[\text{B-int2}]\cdot[\text{P-int2}] - k_{-s1}\cdot[\text{PB-int}_{\text{s}}] - k_{+r}\cdot[\text{PB-int}_{\text{s}}] + k_{-r}\cdot[\text{LR-int}_{\text{s1}}] \tag{13'}$$

$$\frac{d[\text{LR-int}_{\text{s1}}]}{dt} = k_{+r}\cdot[\text{PB-int}_{\text{s}}] - k_{-r}\cdot[\text{LR-int}_{\text{s1}}] - k_{+\text{mod}}\cdot[\text{LR-int}_{\text{s1}}] + k_{-\text{mod}}\cdot[\text{LR-int}_{\text{s2}}] \tag{14'}$$

$$\frac{d[\text{LR-int}_{\text{s2}}]}{dt} = k_{+\text{mod}}\cdot[\text{LR-int}_{\text{s1}}] - k_{-\text{mod}}\cdot[\text{LR-int}_{\text{s2}}] - k_{+s2}\cdot[\text{LR-int}_{\text{s2}}] + k_{-s2}\cdot[\text{L-int2}]\cdot[\text{R-int2}] \tag{15'}$$

$$\frac{d[\text{B-int2-rdf}]}{dt} = k_{+}\cdot[\text{int2-rdf}]\cdot[\text{B}] - k_{+b4}\cdot[\text{B-int2-rdf}] + k_{+}\cdot[\text{rdf}]\cdot([\text{B-int2}] - [\text{B-int2-rdf}]) - k_{-ir}\cdot([\text{B-int2-rdf}] - [\text{B-int2-rdf2}]) - k_{+s0}\cdot[\text{B-int2-rdf}]\cdot([\text{P-int2}]+[\text{P-int2-rdf}]+[\text{P-int2-rdf2}]) + k_{-s02}\cdot([\text{PB-int4-rdf}]+[\text{PB-int4-rdf2}]+[\text{PB-int4-rdf3}]) \tag{16'}$$

$$\frac{d[\text{P-int2-rdf}]}{dt} = k_{+}\cdot[\text{int2-rdf}]\cdot[\text{P}] - k_{+b4}\cdot[\text{P-int2-rdf}] + k_{+}\cdot[\text{rdf}]\cdot([\text{P-int2}] - [\text{P-int2-rdf}]) - k_{-ir}\cdot([\text{P-int2-rdf}] - [\text{P-int2-rdf2}]) - k_{+s0}\cdot[\text{P-int2-rdf}]\cdot([\text{B-int2}]+[\text{B-int2-rdf}]+[\text{B-int2-rdf2}]) + k_{-s02}\cdot([\text{PB-int4-rdf}]+[\text{PB-int4-rdf2}]+[\text{PB-int4-rdf3}]) \tag{17'}$$

$$\frac{d[\text{L-int2-rdf}]}{dt} = k_{+}\cdot[\text{int2-rdf}]\cdot[\text{L}] - k_{-b3}\cdot[\text{L-int2-rdf}] + k_{+}\cdot[\text{rdf}]\cdot([\text{L-int2}] - [\text{L-int2-rdf}]) - k_{-ir}\cdot([\text{L-int2-rdf}] - [\text{L-int2-rdf2}]) - k_{+s0}\cdot[\text{L-int2-rdf}]\cdot([\text{R-int2}]+[\text{R-int2-rdf}]+[\text{R-int2-rdf2}]) + k_{-s01}\cdot([\text{LR-int4-rdf}]+[\text{LR-int4-rdf2}]+[\text{LR-int4-rdf3}]) \tag{18'}$$

$$\frac{d[\text{R-int2-rdf}]}{dt} = k_{+}\cdot[\text{int2-rdf}]\cdot[\text{R}] - k_{-b3}\cdot[\text{R-int2-rdf}] + k_{+}\cdot[\text{rdf}]\cdot([\text{R-int2}] - [\text{R-int2-rdf}]) - k_{-ir}\cdot([\text{R-int2-rdf}] - [\text{R-int2-rdf2}]) - k_{+s0}\cdot[\text{R-int2-rdf}]\cdot([\text{L-int2}]+[\text{L-int2-rdf}]+[\text{L-int2-rdf2}]) + k_{-s01}\cdot([\text{LR-int4-rdf}]+[\text{LR-int4-rdf2}]+[\text{LR-int4-rdf3}]) \tag{19'}$$

$$\frac{d[\text{P-int2-rdf2}]}{dt} = k_{+}\cdot[\text{int2-rdf2}]\cdot[\text{P}] - k_{+b4}\cdot[\text{P-int2-rdf2}] + k_{+}\cdot[\text{rdf}]\cdot[\text{P-int2-rdf}] - k_{-ir}\cdot[\text{P-int2-rdf2}] - k_{-s4}\cdot[\text{P-int2-rdf2}]\cdot[\text{B-int2-rdf2}] + k_{+s4}\cdot[\text{PB-int-rdf}_{\text{s2}}] - k_{+s02}\cdot[\text{P-int2-rdf}]\cdot([\text{B-int2}]+[\text{B-int2-rdf}]) + k_{-s02}\cdot([\text{PB-int4-rdf2}]+[\text{PB-int4-rdf3}]) \qquad (20’)$$

$$\frac{d[\text{B-int2-rdf2}]}{dt} = k_{+}\cdot[\text{int2-rdf2}]\cdot[\text{B}] - k_{+b4}\cdot[\text{B-int2-rdf2}] + k_{+}\cdot[\text{rdf}]\cdot[\text{B-int2-rdf}] - k_{-ir}\cdot[\text{B-int2-rdf2}] - k_{-s4}\cdot[\text{B-int2-rdf2}]\cdot[\text{P-int2-rdf2}] + k_{+s4}\cdot[\text{PB-int-rdf}_{\text{s2}}] - k_{+s02}\cdot[\text{B-int2-rdf}]\cdot([\text{P-int2}]+[\text{P-int2-rdf}]) + k_{-s02}\cdot([\text{PB-int4-rdf2}]+[\text{PB-int4-rdf3}]) \qquad (21’)$$

$$\frac{d[\text{L-int2-rdf2}]}{dt} = k_{+}\cdot[\text{int2-rdf2}]\cdot[\text{L}] - k_{-b3}\cdot[\text{L-int2-rdf2}] + k_{+}\cdot[\text{rdf}]\cdot[\text{L-int2-rdf}] - k_{-ir}\cdot[\text{L-int2-rdf2}] - k_{+s}\cdot[\text{L-int2-rdf2}]\cdot[\text{R-int2-rdf2}] + k_{-s3}\cdot[\text{LR-int-rdf}_{\text{s}}] - k_{+s01}\cdot[\text{L-int2-rdf2}]\cdot([\text{R-int2}]+[\text{R-int2-rdf}]) + k_{-s01}\cdot([\text{LR-int4-rdf2}]+[\text{LR-int4-rdf3}]) \qquad (22’)$$

$$\frac{d[\text{R-int2-rdf2}]}{dt} = k_{+}\cdot[\text{int2-rdf2}]\cdot[\text{R}] - k_{-b3}\cdot[\text{R-int2-rdf2}] + k_{+}\cdot[\text{rdf}]\cdot[\text{R-int2-rdf}] - k_{-ir}\cdot[\text{R-int2-rdf2}] - k_{+s}\cdot[\text{L-int2-rdf2}]\cdot[\text{R-int2-rdf2}] + k_{-s3}\cdot[\text{LR-int-rdf}_{\text{s}}] - k_{+s01}\cdot[\text{R-int2-rdf2}]\cdot([\text{L-int2}]+[\text{L-int2-rdf}]) + k_{-s01}\cdot([\text{LR-int4-rdf2}]+[\text{LR-int4-rdf3}]) \qquad (23’)$$

$$\frac{d[\text{PB-int-rdf}_{\text{s1}}]}{dt} = k_{+r}\cdot[\text{LR-int-rdf}_{\text{s}}] - k_{-r}\cdot[\text{PB-int-rdf}_{\text{s1}}] - k_{+\text{mod}}\cdot[\text{PB-int-rdf}_{\text{s1}}] + k_{-\text{mod}\,r}\cdot[\text{PB-int-rdf}_{\text{s2}}] \qquad (24’)$$

$$\frac{d[\text{PB-int-rdf}_{\text{s2}}]}{dt} = k_{-s4}\cdot[\text{B-int2-rdf2}]\cdot[\text{P-int2-rdf2}] - k_{+s4}\cdot[\text{PB-int-rdf}_{\text{s2}}] + k_{+\text{mod}}\cdot[\text{PB-int-rdf}_{\text{s1}}] - k_{-\text{mod}\,r}\cdot[\text{PB-int-rdf}_{\text{s2}}] \qquad (25’)$$

$$\frac{d[\text{LR-int-rdf}_{\text{s}}]}{dt} = k_{+s}\cdot[\text{L-int2-rdf2}]\cdot[\text{R-int2-rdf2}] - k_{-s3}\cdot[\text{LR-int-rdf}_{\text{s}}] - k_{+r}\cdot[\text{LR-int-rdf}_{\text{s}}] + k_{-r}\cdot[\text{PB-int-rdf}_{\text{s1}}] \qquad (26’)$$

$$\frac{d[\text{B}]}{dt} = k_{-b1}\cdot[\text{B-int2}] + k_{+b4}\cdot([\text{B-int2-rdf}]+[\text{B-int2-rdf2}]) - k_{+}\cdot[\text{B}]\cdot([\text{int2}]+[\text{int2-rdf}]+[\text{int2-rdf2}]) \qquad (27’)$$

$$\frac{d[\text{P}]}{dt} = k_{-b1}\cdot[\text{P-int2}] + k_{+b4}\cdot([\text{P-int2-rdf}]+[\text{P-int2-rdf2}]) - k_{+}\cdot[\text{P}]\cdot([\text{int2}]+[\text{int2-rdf}]+[\text{int2-rdf2}]) \qquad (28’)$$

$$\frac{d[\text{PB-int4-rdf}]}{dt} = k_{+s0}\cdot([\text{P-int2}]\cdot[\text{B-int2-rdf}]+[\text{B-int2}]\cdot[\text{P-int2-rdf}]) - 2k_{-s02}\cdot[\text{PB-int4-rdf}] \qquad (29’)$$

$$\frac{d[\text{PB-int4-rdf2}]}{dt} = k_{+s0}\cdot([\text{P-int2-rdf}]\cdot[\text{B-int2-rdf}]+[\text{B-int2}]\cdot[\text{P-int2-rdf2}]+[\text{P-int2}]\cdot[\text{B-int2-rdf2}]) - 3k_{-s02}\cdot[\text{PB-int4-rdf}] \qquad (30’)$$

$$\frac{d[\text{PB-int4-rdf3}]}{dt} = k_{+s0}\cdot([\text{P-int2-rdf2}]\cdot[\text{B-int2-rdf}]+[\text{B-int2-rdf2}]\cdot[\text{P-int2-rdf}]) - 2k_{-s02}\cdot[\text{PB-int4-rdf}] \qquad (31’)$$

$$\frac{d[\text{LR-int4-rdf}]}{dt} = k_{+s0}\cdot([\text{L-int2}]\cdot[\text{R-int2-rdf}]+[\text{R-int2}]\cdot[\text{L-int2-rdf}]) - 2k_{-s01}\cdot[\text{LR-int4-rdf}] \qquad (32’)$$

$$\frac{d[\text{LR-int4-rdf2}]}{dt} = k_{+s0}\cdot([\text{L-int2-rdf}]\cdot[\text{R-int2-rdf}]+[\text{L-int2}]\cdot[\text{R-int2-rdf2}]+[\text{R-int2}]\cdot[\text{L-int2-rdf2}]) - 3k_{-s01}\cdot[\text{LR-int4-rdf}] \qquad (33’)$$

$$\frac{d[\text{BP-int4-rdf3}]}{dt} = k_{+s0}\cdot([\text{L-int2-rdf2}]\cdot[\text{R-int2-rdf}]+[\text{R-int2-rdf2}]\cdot[\text{L-int2-rdf}]) - 2k_{-s01}\cdot[\text{LR-int4-rdf}] \qquad (34’)$$

$$\frac{d[\text{B-int4}_\text{i}]}{dt} = k_{+i}\cdot[\text{int2}]\cdot[\text{B-int2}] - k_{-i}\cdot[\text{B-int4}_\text{i}] \quad (35')$$

$$\frac{d[\text{P-int4}_\text{i}]}{dt} = k_{+i}\cdot[\text{int2}]\cdot[\text{P-int2}] - k_{-i}\cdot[\text{P-int4}_\text{i}] \quad (36')$$

Parameter values for reactions with linear DNA were chosen to fit our data (Fig. 7) and are presented in supplementary table S2. The relationships between parameters satisfy the energy conservation equations, similarly to the plasmid system:

$$K_{b1}^2\cdot K_{s1}\cdot K_{r1}\cdot K_{\text{mod}}\cdot K_{s2}\cdot K_{b2}^2 = 1;\ K_{b3}^2\cdot K_{s3}\cdot K_{r2}\cdot K_{\text{mod}r}\cdot K_{s4}\cdot K_{b4}^2 = 1 \quad (37')$$

S1.2.3 Model 0 description

The kinetics of Model 0 for plasmid substrates (Fig. 1C), which was used in the development of Model 1, are described by the following system of ODEs:

$$\frac{d[\text{LR}]}{dt} = k_{+b2}\cdot[\text{LR-int2}] - k_{-b2}\cdot[\text{LR}]\cdot[\text{int2}] + k_{-b3}\cdot([\text{LR-int2-rdf}] + [\text{LR-int2-rdf2}]) - k_{+}\cdot[\text{LR}]\cdot([\text{int2-rdf}] + [\text{int2-rdf2}]) \quad (1'')$$

$$\frac{d[\text{int}]}{dt} = k_{-ii}\cdot(2\cdot[\text{int2}] + [\text{int2-rdf}]) + k_{-ir}\cdot[\text{int-rdf}] - k_{+}\cdot(2\cdot[\text{int}]^2 + [\text{int}]\cdot[\text{int-rdf}] + [\text{int}]\cdot[\text{rdf}]) \quad (2'')$$

$$\frac{d[\text{int-rdf}]}{dt} = k_{+}\cdot([\text{int}]\cdot[\text{rdf}] - [\text{int}]\cdot[\text{int-rdf}] - 2\cdot[\text{int-rdf}]^2) - k_{-ir}\cdot[\text{int-rdf}] + k_{-ii}\cdot([\text{int2-rdf}] + 2\cdot[\text{int2-rdf2}]) \quad (3'')$$

$$\frac{d[\text{int2}]}{dt} = k_{+}\cdot[\text{int}]^2 - k_{-ii}\cdot[\text{int2}] - k_{+}\cdot[\text{int2}]\cdot([\text{rdf}] + [\text{PB}] + [\text{PB-int2}]) - k_{-b2}\cdot[\text{int2}]\cdot([\text{LR}] + [\text{LR-int2}]) - k_{+s0}\cdot[\text{int2}]\cdot([\text{LR-int2-rdf}] + [\text{LR-int2-rdf2}] + [\text{PB-int2-rdf}] + [\text{PB-int2-rdf2}]) + k_{-ir}\cdot[\text{int2-rdf}] + k_{-b1}\cdot([\text{BP-int2}] + [\text{PB-int4}]) + k_{-s01}\cdot([\text{LR-int4-rdf}] + [\text{LR-int4-rdf2}]) + k_{-s02}\cdot([\text{PB-int4-rdf}] + [\text{PB-int4-rdf2}]) + k_{+b2}\cdot([\text{LR-int2}] + [\text{LR-int4}]) \quad (4'')$$

$$\frac{d[\text{int2-rdf}]}{dt} = k_{+}\cdot([\text{int2}]\cdot[\text{rdf}] + [\text{int}]\cdot[\text{int-rdf}]) - (k_{-ir} + k_{-ii})\cdot[\text{int2-rdf}] - k_{+}\cdot[\text{int2-rdf}]\cdot[\text{rdf}] + k_{-ir}\cdot[\text{int2-rdf2}] - k_{+}\cdot[\text{int2-rdf}]\cdot([\text{PB}] + [\text{LR}]) - k_{+s0}\cdot[\text{int2-rdf}]\cdot([\text{LR-int2}] + [\text{LR-int2-rdf}] + [\text{LR-int2-rdf2}] + [\text{PB-int2}] + [\text{PB-int2-rdf}] + [\text{PB-int2-rdf2}]) + k_{+b4}\cdot[\text{PB-int2-rdf}] + k_{-s01}\cdot([\text{LR-int4-rdf}] + [\text{LR-int4-rdf2}] + [\text{LR-int4-rdf3}]) + k_{-s02}\cdot([\text{PB-int4-rdf}] + [\text{PB-int4-rdf2}] + [\text{PB-int4-rdf3}]) + k_{-b3}\cdot[\text{LR-int2-rdf}] \quad (5'')$$

$$\frac{d[\text{int2-rdf2}]}{dt} = k_{+}\cdot([\text{int2-rdf}]\cdot[\text{rdf}] + [\text{int-rdf}]^2) - (k_{-ir} + k_{-ii})\cdot[\text{int2-rdf2}] - k_{+}\cdot[\text{int2-rdf2}]\cdot([\text{PB}] + [\text{LR}] + [\text{PB-int2-rdf2}] + [\text{LR-int2-rdf2}]) - k_{+s01}\cdot[\text{int2-rdf2}]\cdot([\text{LR-int2}] + [\text{LR-int2-rdf}] + ([\text{PB-int2}] + [\text{PB-int2-rdf}]) + k_{+b4}\cdot([\text{PB-int2-rdf2}] + [\text{PB-int4-rdf4}]) + k_{-s01}\cdot([\text{LR-int4-rdf2}] + [\text{LR-int4-rdf3}]) + k_{-s02}\cdot([\text{PB-int4-rdf2}] + [\text{PB-int4-rdf3}]) + k_{-b3}\cdot([\text{LR-int2-rdf2}] + [\text{LR-int4-rdf4}]) \quad (6'')$$

$$\frac{d[\text{rdf}]}{dt} = k_{-ir}\cdot([\text{int-rdf}] + [\text{int2-rdf}] + [\text{int2-rdf2}] + [\text{PB-int2-rdf}] + [\text{LR-int2-rdf}] + [\text{PB-int2-rdf2}] + [\text{LR-int2-rdf2}]) - k_{+}\cdot[\text{rdf}]\cdot([\text{int}] + [\text{int2}] + [\text{int2-rdf}] + [\text{PB-int2}] + [\text{LR-int2}] + [\text{PB-int2-rdf}] + [\text{LR-int2-rdf}])$$

(7'')

$$\frac{d[\mathrm{PB\text{-}int2}]}{dt}=k_{+}\cdot[\mathrm{int2}]\cdot[\mathrm{PB}]-k_{-b1}\cdot[\mathrm{PB\text{-}int2}]-k_{+}\cdot[\mathrm{PB\text{-}int2}]\cdot[\mathrm{int2}]-k_{+s0}\cdot[\mathrm{PB\text{-}int2}]\cdot ([\mathrm{int2\text{-}rdf}]+[\mathrm{int2\text{-}rdf2}])+k_{-b1}\cdot[\mathrm{PB\text{-}int4}]+k_{-s02}\cdot([\mathrm{PB\text{-}int4\text{-}rdf}]+[\mathrm{PB\text{-}int4\text{-}rdf2}])- k_{+}\cdot[\mathrm{rdf}]\cdot[\mathrm{PB\text{-}int2}]-k_{-ir}\cdot[\mathrm{PB\text{-}int2\text{-}rdf}] \quad (8'')$$

$$\frac{d[\mathrm{LR\text{-}int2}]}{dt}=k_{-b2}\cdot[\mathrm{int2}]\cdot([\mathrm{LR}]-[\mathrm{LR\text{-}int2}])-k_{+b2}\cdot([\mathrm{LR\text{-}int2}]-[\mathrm{LR\text{-}int4}])-k_{+s0}\cdot[\mathrm{LR\text{-}int2}]\cdot([\mathrm{int2\text{-}rdf}] +[\mathrm{int2\text{-}rdf2}])+k_{-s01}\cdot([\mathrm{LR\text{-}int4\text{-}rdf}]+[\mathrm{LR\text{-}int4\text{-}rdf2}])-k_{+}\cdot[\mathrm{rdf}]\cdot[\mathrm{LR\text{-}int2}]+k_{+ir}\cdot[\mathrm{LR\text{-}int2\text{-}rdf}] \quad (9'')$$

$$\frac{d[\mathrm{PB\text{-}int4}]}{dt}=k_{+}\cdot[\mathrm{int2}]\cdot[\mathrm{PB\text{-}int2}]-k_{-b1}\cdot[\mathrm{PB\text{-}int4}]-k_{+s1}\cdot[\mathrm{PB\text{-}int4}]+k_{-s1}\cdot[\mathrm{PB\text{-}int_s}] \quad (10'')$$

$$\frac{d[\mathrm{LR\text{-}int4}]}{dt}=k_{-b2}\cdot[\mathrm{int2}]\cdot[\mathrm{LR\text{-}int2}]-k_{+b2}\cdot[\mathrm{LR\text{-}int4}]-k_{+s1}\cdot[\mathrm{LR\text{-}int4}]+k_{-s1}\cdot[\mathrm{LR\text{-}int_{s1}}] \quad (11'')$$

$$\frac{d[\mathrm{PB\text{-}int_s}]}{dt}=k_{+s1}\cdot[\mathrm{PB\text{-}int4}]-k_{-s1}\cdot[\mathrm{PB\text{-}int_s}]-k_{+r}\cdot[\mathrm{PB\text{-}int_s}]+k_{-r}\cdot[\mathrm{LR\text{-}int_{s1}}] \quad (12'')$$

$$\frac{d[\mathrm{LR\text{-}int_{s1}}]}{dt}=k_{+r}\cdot[\mathrm{PB\text{-}int_s}]-k_{-r}\cdot[\mathrm{LR\text{-}int_{s1}}]+k_{+s1}\cdot[\mathrm{LR\text{-}int4}]-k_{-s1}\cdot[\mathrm{LR\text{-}int_{s1}}] \quad (13'')$$

$$\frac{d[\mathrm{PB\text{-}int2\text{-}rdf}]}{dt}=k_{+}\cdot[\mathrm{int2\text{-}rdf}]\cdot[\mathrm{PB}]-k_{+b4}\cdot[\mathrm{PB\text{-}int2\text{-}rdf}]-k_{+s0}\cdot[\mathrm{PB\text{-}int2\text{-}rdf}]\cdot([\mathrm{int2}]+ [\mathrm{int2\text{-}rdf}]+[\mathrm{int2\text{-}rdf2}])+k_{-s02}\cdot([\mathrm{PB\text{-}int4\text{-}rdf}]+[\mathrm{PB\text{-}int4\text{-}rdf2}]+[\mathrm{PB\text{-}int4\text{-}rdf3}])+ k_{+}\cdot[\mathrm{rdf}]\cdot([\mathrm{PB\text{-}int2}]-[\mathrm{PB\text{-}int2\text{-}rdf}])-k_{-ir}\cdot([\mathrm{PB\text{-}int2\text{-}rdf}]-[\mathrm{PB\text{-}int2\text{-}rdf2}]) \quad (14'')$$

$$\frac{d[\mathrm{LR\text{-}int2\text{-}rdf}]}{dt}=k_{+}\cdot[\mathrm{int2\text{-}rdf}]\cdot[\mathrm{LR}]-k_{-b3}\cdot[\mathrm{LR\text{-}int2\text{-}rdf}]-k_{+s0}\cdot[\mathrm{LR\text{-}int2\text{-}rdf}]\cdot([\mathrm{int2}]+ [\mathrm{int2\text{-}rdf}]+[\mathrm{int2\text{-}rdf2}])+k_{-s01}\cdot([\mathrm{LR\text{-}int4\text{-}rdf}]+[\mathrm{LR\text{-}int4\text{-}rdf2}]+[\mathrm{LR\text{-}int4\text{-}rdf3}])+ k_{+}\cdot[\mathrm{rdf}]\cdot([\mathrm{LR\text{-}int2}]-[\mathrm{LR\text{-}int2\text{-}rdf}])-k_{-ir}\cdot([\mathrm{LR\text{-}int2\text{-}rdf}]-[\mathrm{LR\text{-}int2\text{-}rdf2}]) \quad (15'')$$

$$\frac{d[\mathrm{PB\text{-}int2\text{-}rdf2}]}{dt}=k_{+}\cdot[\mathrm{int2\text{-}rdf2}]\cdot[\mathrm{PB}]-k_{+b4}\cdot[\mathrm{PB\text{-}int2\text{-}rdf2}]+k_{+}\cdot[\mathrm{PB\text{-}int2\text{-}rdf}]\cdot[\mathrm{rdf}]- k_{+ir}\cdot[\mathrm{PB\text{-}int2\text{-}rdf2}]-k_{+}\cdot[\mathrm{PB\text{-}int2\text{-}rdf2}]\cdot[\mathrm{int2\text{-}rdf2}]+k_{+b4}\cdot[\mathrm{PB\text{-}int4\text{-}rdf4}]-k_{+s0}\cdot[\mathrm{PB\text{-}int2\text{-}rdf2}]\cdot ([\mathrm{int2}]+[\mathrm{int2\text{-}rdf}])+k_{-s02}\cdot([\mathrm{PB\text{-}int4\text{-}rdf2}]+[\mathrm{PB\text{-}int4\text{-}rdf3}]) \quad (16'')$$

$$\frac{d[\mathrm{LR\text{-}int2\text{-}rdf2}]}{dt}=k_{+}\cdot[\mathrm{int2\text{-}rdf2}]\cdot[\mathrm{LR}]-k_{-b3}\cdot[\mathrm{LR\text{-}int2\text{-}rdf2}]+k_{+}\cdot[\mathrm{LR\text{-}int2\text{-}rdf}]\cdot[\mathrm{rdf}]- k_{+ir}\cdot[\mathrm{LR\text{-}int2\text{-}rdf2}]-k_{+}\cdot[\mathrm{LR\text{-}int2\text{-}rdf2}]\cdot[\mathrm{int2\text{-}rdf2}]+k_{-b3}\cdot[\mathrm{LR\text{-}int4\text{-}rdf4}]-k_{+s0}\cdot[\mathrm{LR\text{-}int2\text{-}rdf2}]\cdot ([\mathrm{int2}]+[\mathrm{int2\text{-}rdf}])+k_{-s01}\cdot([\mathrm{LR\text{-}int4\text{-}rdf2}]+[\mathrm{LR\text{-}int4\text{-}rdf3}]) \quad (17'')$$

$$\frac{d[\mathrm{PB\text{-}int4\text{-}rdf4}]}{dt}=k_{+}\cdot[\mathrm{int2\text{-}rdf2}]\cdot[\mathrm{PB\text{-}int2\text{-}rdf2}]-k_{+b4}\cdot[\mathrm{PB\text{-}int4\text{-}rdf4}]- k_{+s3}\cdot[\mathrm{PB\text{-}int4\text{-}rdf4}]+k_{-s3}\cdot[\mathrm{PB\text{-}int\text{-}rdf_{s1}}] \quad (18'')$$

$$\frac{d[\mathrm{LR\text{-}int4\text{-}rdf4}]}{dt}=k_{+}\cdot[\mathrm{int2\text{-}rdf2}]\cdot[\mathrm{LR\text{-}int2\text{-}rdf2}]-k_{-b3}\cdot[\mathrm{LR\text{-}int4\text{-}rdf4}]- k_{+s3}\cdot[\mathrm{LR\text{-}int4\text{-}rdf4}]+k_{-s3}\cdot[\mathrm{LR\text{-}int\text{-}rdf_s}] \quad (19'')$$

$$\frac{d[\mathrm{PB\text{-}int\text{-}rdf_{s1}}]}{dt}=k_{+r}\cdot[\mathrm{LR\text{-}int\text{-}rdf_s}]-k_{-r}\cdot[\mathrm{PB\text{-}int\text{-}rdf_{s1}}]- k_{-s3}\cdot[\mathrm{PB\text{-}int\text{-}rdf_{s1}}]+k_{+s3}\cdot[\mathrm{PB\text{-}int4\text{-}rdf4}] \quad (20'')$$

$$\frac{d[\mathrm{LR\text{-}int\text{-}rdf_s}]}{dt}=k_{+s3}\cdot[\mathrm{LR\text{-}int4\text{-}rdf4}]-k_{-s3}\cdot[\mathrm{LR\text{-}int\text{-}rdf_s}]- k_{+r}\cdot[\mathrm{LR\text{-}int\text{-}rdf_s}]+k_{-r}\cdot[\mathrm{BP\text{-}int\text{-}rdf_{s1}}] \quad (21'')$$

$$\frac{d[\mathrm{PB}]}{dt} = k_{-b1} \cdot [\mathrm{PB\text{-}int2}] + k_{+b4} \cdot ([\mathrm{PB\text{-}int2\text{-}rdf}] + [\mathrm{PB\text{-}int2\text{-}rdf2}]) - k_{+} \cdot [\mathrm{PB}] \cdot ([\mathrm{int2}] + [\mathrm{int2\text{-}rdf}] + [\mathrm{int2\text{-}rdf2}]) \quad (22'')$$

$$\frac{d[\mathrm{PB\text{-}int4\text{-}rdf}]}{dt} = k_{+s0} \cdot ([\mathrm{int2}] \cdot [\mathrm{PB\text{-}int2\text{-}rdf}] + [\mathrm{int2\text{-}rdf}] \cdot [\mathrm{PB\text{-}int2}]) - 2k_{-s02} \cdot [\mathrm{PB\text{-}int4\text{-}rdf}] \quad (23'')$$

$$\frac{d[\mathrm{PB\text{-}int4\text{-}rdf2}]}{dt} = k_{+s0} \cdot ([\mathrm{int2}] \cdot [\mathrm{PB\text{-}int2\text{-}rdf2}] + [\mathrm{int2\text{-}rdf}] \cdot [\mathrm{PB\text{-}int2\text{-}rdf}] + [\mathrm{int2\text{-}rdf2}] \cdot [\mathrm{PB\text{-}int2}]) - 3k_{-s02} \cdot [\mathrm{PB\text{-}int4\text{-}rdf2}] \quad (24'')$$

$$\frac{d[\mathrm{PB\text{-}int4\text{-}rdf3}]}{dt} = k_{+s0} \cdot ([\mathrm{int2\text{-}rdf}] \cdot [\mathrm{PB\text{-}int2\text{-}rdf2}] + [\mathrm{int2\text{-}rdf2}] \cdot [\mathrm{PB\text{-}int2\text{-}rdf}]) - 2k_{-s02} \cdot [\mathrm{PB\text{-}int4\text{-}rdf3}] \quad (25'')$$

$$\frac{d[\mathrm{LR\text{-}int4\text{-}rdf}]}{dt} = k_{+s0} \cdot ([\mathrm{int2}] \cdot [\mathrm{LR\text{-}int2\text{-}rdf}] + [\mathrm{int2\text{-}rdf}] \cdot [\mathrm{LR\text{-}int2}]) - 2k_{-s01} \cdot [\mathrm{LR\text{-}int4\text{-}rdf}] \quad (26'')$$

$$\frac{d[\mathrm{LR\text{-}int4\text{-}rdf2}]}{dt} = k_{+s0} \cdot ([\mathrm{int2}] \cdot [\mathrm{LR\text{-}int2\text{-}rdf2}] + [\mathrm{int2\text{-}rdf}] \cdot [\mathrm{LR\text{-}int2\text{-}rdf}] + [\mathrm{int2\text{-}rdf2}] \cdot [\mathrm{LR\text{-}int2}]) - 3k_{-s01} \cdot [\mathrm{LR\text{-}int4\text{-}rdf2}] \quad (27'')$$

$$\frac{d[\mathrm{LR\text{-}int4\text{-}rdf3}]}{dt} = k_{+s0} \cdot ([\mathrm{int2\text{-}rdf}] \cdot [\mathrm{LR\text{-}int2\text{-}rdf2}] + [\mathrm{int2\text{-}rdf2}] \cdot [\mathrm{LR\text{-}int2\text{-}rdf}]) - 2k_{-s01} \cdot [\mathrm{LR\text{-}int4\text{-}rdf3}] \quad (28'')$$

The parameter values were chosen to fit the data for the permitted reactions (Fig. 5).

The equations for the conservation of energies during conversion of PB to LR and LR to PB are:

$$K_{b1}^{2} \cdot K_{s1} \cdot K_{r1} \cdot K_{s2} \cdot K_{b2}^{2} = 1;\ K_{b3}^{2} \cdot K_{s3} \cdot K_{r2} \cdot K_{s4} \cdot K_{b4}^{2} = 1 \quad (29'')$$

The values of the Model 0 parameters are presented in Table S3.

S1.3 References

1. Chambers, S.P., Prior, S.E., Barstow, D.A. and Minton, N.P. (1988) The pMTL nic-cloning vectors. I. Improved pUC polylinker regions to facilitate the use of sonicated DNA for nucleotide sequencing. *Gene*, **68**, 139-149.
2. Khaleel, T., Younger, E., McEwan, A.R., Varghese, A.S. and Smith, M.C. (2011) A phage protein that binds ϕC31 integrase to switch its directionality. *Mol Microbiol*, **80**, 1450-1463.
3. Grindley, N.D., Whiteson, K.L. and Rice, P.A. (2006) Mechanisms of site-specific recombination. *Annu Rev Biochem*, **75**, 567-605.
4. Ringrose, L., Lounnas, V., Ehrlich, L., Buchholz, F., Wade, R. and Stewart, A.F. (1998) Comparative kinetic analysis of FLP and Cre recombinases: mathematical models for DNA binding and recombination. *J Mol Biol*, **284**, 363-384.
5. Schreiber, G. (2002) Kinetic studies of protein-protein interactions. *Curr Opin Struct Biol*, **12**, 41-47.

S1.4 Matlab code of Model 1 for plasmid substrates

All Matlab code is available on Github at https://github.com/QTB-HHU/integraseModel
Code for Model 1 for plasmids is shown below.

```
Kr1=1; Kr2=1; Dtot=0.01; Ki=0.02;
Ks1=0.1; Ks2=0.12; Ks3=0.1; Ks4=0.013;
Kii=0.3; Kir=0.05; Kmod=3.4; Kmodr=1.9;
Kb1=0.02; Kb2=0.01; Kb3=0.025; Kb4=0.05; % Kb1, Kb3, Ks1, Ks3 – dissociation constants

% time unit - min

% y(1)   LR
% y(2)   int
% y(3)   int2-rdf2
% y(4)   int2
% y(5)   PB-int2
% y(6)   LR-int2
% y(7)   PB-int4
% y(8)   LR-int4
% y(9)   LR-int4 synapse second
% y(10)  PB-int4 synapse
% y(11)  LR-int4 synapse first
% y(12)  PB-int2-rdf2
% y(13)  LR-int2-rdf2
% y(14)  PB-int4-rdf4
% y(15)  LR-int4-rdf4
% y(16)  PB-int4-rdf4 synapse second
% y(17)  PB-int4-rdf4 synapse first
% y(18)  LR-int4-rdf4 synapse
% y(19)  int-rdf
% y(20)  int2-rdf
% y(21)  PB-int2-rdf
% y(22)  LR-int2-rdf
% y(23)  rdf
% y(24)  PB
% y(25)  PB-int4-rdf
% y(26)  PB-int4-rdf2
% y(27)  PB-int4-rdf3
% y(28)  LR-int4-rdf
% y(29)  LR-int4-rdf2
% y(30)  LR-int4-rdf3
% y(31)  PB-int6i
% y(32)  PB-int6-rdf4i
% y(33)
% y(34)  PB-int6-rdfi
% y(35)  PB-int6-rdf2i
% y(36)  PB-int6-rdf3i

y0=[0 0.4 0 0 0 0 0 0 0 0 0 0 0 0 0 0 0 0 0 0 0 0 0. Dtot 0 0 0 0 0 0 0 0 0 0 0 0];
%y0=[Dtot 0.4 0 0 0 0 0 0 0 0 0 0 0 0 0 0 0 0 0 0 0 0 0.8 0 0 0 0 0 0 0 0 0 0 0 0 0];

tJ=[0 1 2 4 8 16 32 64 120 180]; % kinetics int 400 nM;  DNA=10 nM
prJ1=[0 28 36 47 56 64 68 72 73 74]; % 0.4 int + PB
pr_err1=[0 2 1 1 2 2 1 2 2 2];
prJ2=[0 24 32 42 51 57 61 64 66 67]; % 0.4 int + 0.8 rdf + LR
pr_err2=[0 3 2 1 1 1 1 1 2 2];

options = odeset();

    t=[0 180];
    [T, Y] = 
ode15s(@Model_integrase_f,t,y0,options,Kii,Ki,Kir,Kb1,Kb2,Kb3,Kb4,Ks1,Ks2,Ks3,Ks4,Kr1,Kr2,Kmo
d,Kmodr);

LRt=Y(:,1)+Y(:,6)+Y(:,8)+Y(:,9)+Y(:,11)+Y(:,13)+Y(:,15)+Y(:,18)+Y(:,22)+Y(:,28)+Y(:,29)+Y(:,3
0);
PBt=Y(:,5)+Y(:,7)+Y(:,10)+Y(:,12)+Y(:,14)+Y(:,16)+Y(:,17)+Y(:,21)+Y(:,24)+Y(:,25)+Y(:,26)+Y(:
,27)+Y(:,31)+Y(:,32)+Y(:,34)+Y(:,35)+Y(:,36);
```

```
int_tot=Y(:,2)+Y(:,19)+2*(Y(:,3)+Y(:,20)+Y(:,21)+Y(:,22)+Y(:,4)+Y(:,5)+Y(:,6)+Y(:,12)+Y(:,13)
)+4*(Y(:,7)+Y(:,8)+Y(:,9)+Y(:,10)+Y(:,11)+Y(:,14)+Y(:,15)+Y(:,16)+Y(:,17)+Y(:,18)+Y(:,28)+Y(:
,29)+Y(:,30)+Y(:,25)+Y(:,26)+Y(:,27))+6*(Y(:,31)+Y(:,32)+Y(:,34)+Y(:,35)+Y(:,36));
rdf_tot=Y(:,23)+Y(:,19)+Y(:,20)+Y(:,21)+Y(:,22)+Y(:,25)+Y(:,28)+Y(:,34)+2*(Y(:,3)+Y(:,12)+Y(:
,13)+Y(:,26)+Y(:,29)+Y(:,35))+3*(Y(:,27)+Y(:,30)+Y(:,36))+4*(Y(:,14)+Y(:,15)+Y(:,16)+Y(:,17)+
Y(:,18)+Y(:,32));
DNA_rdf=Y(:,13)+Y(:,15)+Y(:,18)+Y(:,22)+Y(:,28)+Y(:,29)+Y(:,30)+Y(:,12)+Y(:,14)+Y(:,16)+Y(:,1
7)+Y(:,21)+Y(:,25)+Y(:,26)+Y(:,27)+Y(:,32)+Y(:,34)+Y(:,36);

figure (1)
plot(T,LRt/Dtot,'r');
hold on;
plot(T,PBt/Dtot,'b');
hold on;
plot(T,Y(:,9)/Dtot,'r--');
hold on;
plot(T,Y(:,11)/Dtot,'r:');
hold on;
plot(T,Y(:,16)/Dtot,'b--');
hold on;
plot(T,Y(:,17)/Dtot,'b:');
hold on;
title({'LRtot-red (LR-int_s_2 - dash; LR-int_s_1 - dot)';'PBtot-blue (PB-int-rdf_s_2- dash;
PB-int-rdf_s_1- dot)'});

figure (2)
plot(T,PBt/Dtot*100,'b');
hold on;
plot(T,LRt/Dtot*100,'r');
hold on;
errorbar(tJ,prJ1,pr_err1,'r:');
hold on;
errorbar(tJ,prJ2,pr_err2,'b:');
hold on;
title('LRtot(%)-red; PBtot(%)-blue');
```

the program uses the following function (file named Model_integrase_f.m)

```
function Func =
Model_integrase_f(t,y,Kii,Ki,Kir,Kb1,Kb2,Kb3,Kb4,Ks1,Ks2,Ks3,Ks4,Kr1,Kr2,Kmod,Kmodr,Ks01,Ks02
);


Func = zeros(36, 1);

% y(1)   LR
% y(2)   int
% y(3)   int2-rdf2
% y(4)   int2
% y(5)   PB-int2
% y(6)   LR-int2
% y(7)   PB-int4
% y(8)   LR-int4
% y(9)   LR-int4 synapse second
% y(10)  PB-int4 synapse
% y(11)  LR-int4 synapse first
% y(12)  PB-int2-rdf2
% y(13)  LR-int2-rdf2
% y(14)  PB-int4-rdf4
% y(15)  LR-int4-rdf4
% y(16)  PB-int4-rdf4 synapse second
% y(17)  PB-int4-rdf4 synapse first
% y(18)  LR-int4-rdf4 synapse
% y(19)  int-rdf
% y(20)  int2-rdf
% y(21)  PB-int2-rdf
% y(22)  LR-int2-rdf
% y(23)  rdf
% y(24)  PB
% y(25)  PB-int4-rdf
% y(26)  PB-int4-rdf2
% y(27)  PB-int4-rdf3
% y(28)  LR-int4-rdf
% y(29)  LR-int4-rdf2
```

```
% y(30)  LR-int4-rdf3
% y(31)  PB-int6i
% y(32)  PB-int6-rdf4i
% y(33)
% y(34)  PB-int6-rdfi
% y(35)  PB-int6-rdf2i
% y(36)  PB-int6-rdf3i

% kp - association rate; km - dissociation rate

kps01=40;
kps02=kps01;
kp=60;
kmii=Kii*kp;
kmir=Kir*kp;
kmb1=Kb1*kp;
kmb2=10;
kpb2=Kb2*kmb2;
kmb3=Kb3*kp;
kpb4=Kb4*kp;
kpi=3;
kmi=Ki*kpi;
kps1=0.8;
kps3=kps1;
kms01=Ks01*kps01;
kms02=Ks02*kps02;
kms1=Ks1*kps1;
kms3=Ks3*kps3;
kms2=0.0001;
kps2=Ks2*kms2;
kms4=0.005;
kps4=Ks4*kms4;
kpr=1;
kmr1=kpr/Kr1;
kmr2=kpr/Kr2;
kpmod=1;
kpmodr=kpmod;
kmmod=kpmod/Kmod;
kmmodr=kpmodr/Kmodr;

Func(1) = kpb2*y(6)-kmb2*y(1)*y(4)+kmb3*(y(13)+y(22))-kp*y(1)*(y(3)+y(20));
Func(2) = kmii*(2*y(4)+y(20))+kmir*y(19)-kp*(2*y(2)*y(2)+y(2)*y(19)+y(2)*y(23));
Func(3) = kp*(y(20)*y(23)+y(19)*y(19))-(kmir+kmii)*y(3)-kp*y(3)*(y(24)+y(12)+y(1)+y(13))-
kps01*y(3)*(y(6)+y(22))-
kps02*y(3)*(y(5)+y(21))+kpb4*(y(12)+y(14))+kms01*(y(29)+y(30))+kms02*(y(26)+y(27))+kmb3*(y(13
)+y(15));
Func(4) = kp*y(2)*y(2)-kmii*y(4)-kp*y(4)*(y(23)+y(24)+y(5))-
kmb2*y(4)*(y(1)+y(6))+kpb2*(y(6)+y(8))-kps01*y(4)*(y(13)+y(22))-
kps02*y(4)*(y(12)+y(21))+kmir*y(20)+kmb1*(y(5)+y(7))+kms01*(y(28)+y(29))+kms02*(y(25)+y(26))-
(kpi*y(4)*(y(7)+y(14)+y(25)+y(26)+y(27))-kmi*(y(31)+y(32)+y(34)+y(35)+y(36)));
Func(5) = kp*y(24)*y(4)-kmb1*y(5)-kp*y(5)*y(4)-
kps02*y(5)*(y(20)+y(3))+kmb1*y(7)+kms02*(y(25)+y(26))-kp*y(23)*y(5)+kmir*y(21);
Func(6) = kmb2*y(4)*(y(1)-y(6))-kpb2*(y(6)-y(8))-kps01*y(6)*(y(20)+y(3))+kms01*(y(28)+y(29))-
kp*y(23)*y(6)+kmir*y(22);
Func(7) = kp*y(5)*y(4)-kmb1*y(7)-kps1*y(7)+kms1*y(10)-(kpi*y(4)*y(7)-y(31)*kmi);
Func(8) = kmb2*y(6)*y(4)-kpb2*y(8)-kms2*y(8)+kps2*y(9);
Func(9) = kpmod*y(11)-kmmod*y(9)-kps2*y(9)+kms2*y(8);
Func(10) = kps1*y(7)-kms1*y(10)-kpr*y(10)+kmr1*y(11);
Func(11) = kpr*y(10)-kmr1*y(11)-kpmod*y(11)+kmmod*y(9);
Func(12) = kp*y(24)*y(3)-kpb4*y(12)+kp*y(21)*y(23)-kmir*y(12)-kp*y(12)*y(3)-
kps02*y(12)*(y(4)+y(20))+kms02*(y(26)+y(27))+kpb4*y(14);
Func(13) = kp*y(1)*y(3)-kmb3*y(13)+kp*y(22)*y(23)-kmir*y(13)-kp*y(13)*y(3)-
kps01*y(13)*(y(4)+y(20))+kms01*(y(29)+y(30))+kmb3*y(15);
Func(14) = kp*y(3)*y(12)-kpb4*y(14)+kps4*y(16)-kms4*y(14)-(kpi*y(4)*y(14)-y(32)*kmi);
Func(15) = kp*y(3)*y(13)-kmb3*y(15)-kps3*y(15)+kms3*y(18);
Func(16) = kpmodr*y(17)-kmmodr*y(16)-kps4*y(16)+kms4*y(14);
Func(17) = kpr*y(18)-kmr2*y(17)+kmmodr*y(16)-kpmodr*y(17);
Func(18) = kps3*y(15)-kms3*y(18)-kpr*y(18)+kmr2*y(17);
Func(19) = kp*(y(2)*y(23)-y(2)*y(19)-2*y(19)*y(19))-kmir*y(19)+kmii*(y(20)+2*y(3));
Func(20) = kp*(y(4)*y(23)+y(2)*y(19))-(kmir+kmii)*y(20)-kp*y(23)*y(20)+kmir*y(3)-
kp*y(20)*(y(24)+y(1))-kps01*y(20)*(y(6)+y(22)+y(13))-
kps02*y(20)*(y(5)+y(21)+y(12))+kpb4*y(21)+kms01*(y(28)+y(29)+y(30))+kms02*(y(25)+y(26)+y(27))
+kmb3*y(22);
```

```
Func(21) = kp*y(24)*y(20)-kpb4*y(21)-
kps02*y(21)*(y(4)+y(20)+y(3))+kms02*(y(25)+y(26)+y(27))+kp*y(23)*y(5)-kmir*y(21)-
kp*y(21)*y(23)+kmir*y(12);
Func(22) = kp*y(1)*y(20)-kmb3*y(22)-
kps01*y(22)*(y(20)+y(3)+y(4))+kms01*(y(28)+y(29)+y(30))+kp*y(23)*y(6)-kmir*y(22)-
kp*y(22)*y(23)+kmir*y(13);
Func(23) = kmir*(y(19)+y(20)+y(3)+y(21)+y(22)+y(12)+y(13))-
kp*y(23)*(y(2)+y(4)+y(20)+y(5)+y(6)+y(21)+y(22));
Func(24) = kmb1*y(5)+kpb4*(y(12)+y(21))-kp*y(24)*(y(4)+y(20)+y(3));
Func(25) = kps02*(y(4)*y(21)+y(20)*y(5))-y(25)*2*kms02-(kpi*y(4)*y(25)-y(34)*kmi);
Func(26) = kps02*(y(4)*y(12)+y(20)*y(21)+y(3)*y(5))-y(26)*3*kms02-(kpi*y(4)*y(26)-y(35)*kmi);
Func(27) = kps02*(y(20)*y(12)+y(3)*y(21))-y(27)*2*kms02-(kpi*y(4)*y(27)-y(36)*kmi);
Func(28) = kps01*(y(4)*y(22)+y(20)*y(6))-y(28)*2*kms01;
Func(29) = kps01*(y(4)*y(13)+y(20)*y(22)+y(3)*y(6))-y(29)*3*kms01;
Func(30) = kps01*(y(20)*y(13)+y(3)*y(22))-y(30)*2*kms01;
Func(31) = kpi*y(4)*y(7)-y(31)*kmi;
Func(32) = kpi*y(4)*y(14)-y(32)*kmi;
Func(33) = 0;
Func(34) = kpi*y(4)*y(25)-y(34)*kmi;
Func(35) = kpi*y(4)*y(26)-y(35)*kmi;
Func(36) = kpi*y(4)*y(27)-y(36)*kmi;
```

S1.5 Supplementary figures

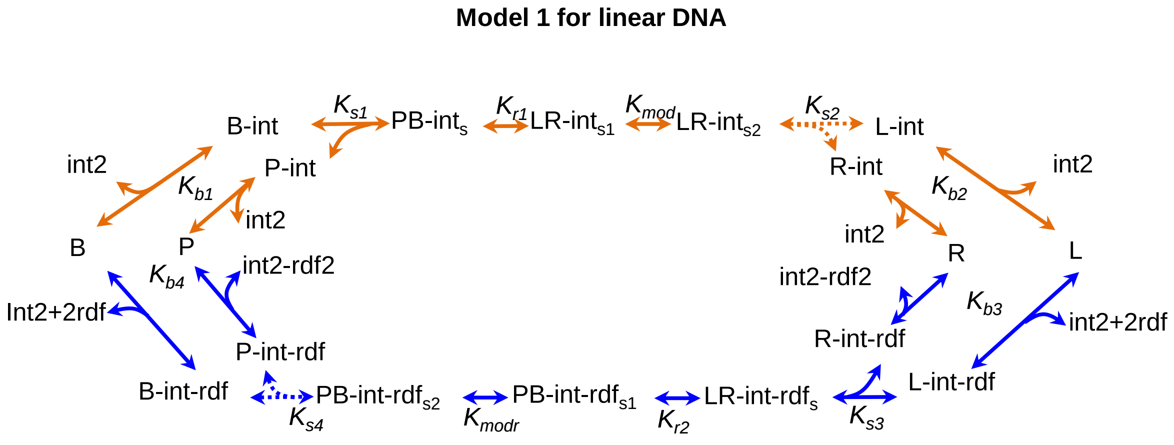

Figure S1. Scheme showing Model 1 as adapted for linear DNA substrates. Reactions in the presence and absence of RDF are shown by orange and blue arrows respectively. Slow steps (dissociation of product synapses) are shown by dotted arrows. Linear DNA molecules containing a single site (*attP*, *attB*, *attL* or *attR*) are represented by P, B, L and R respectively.

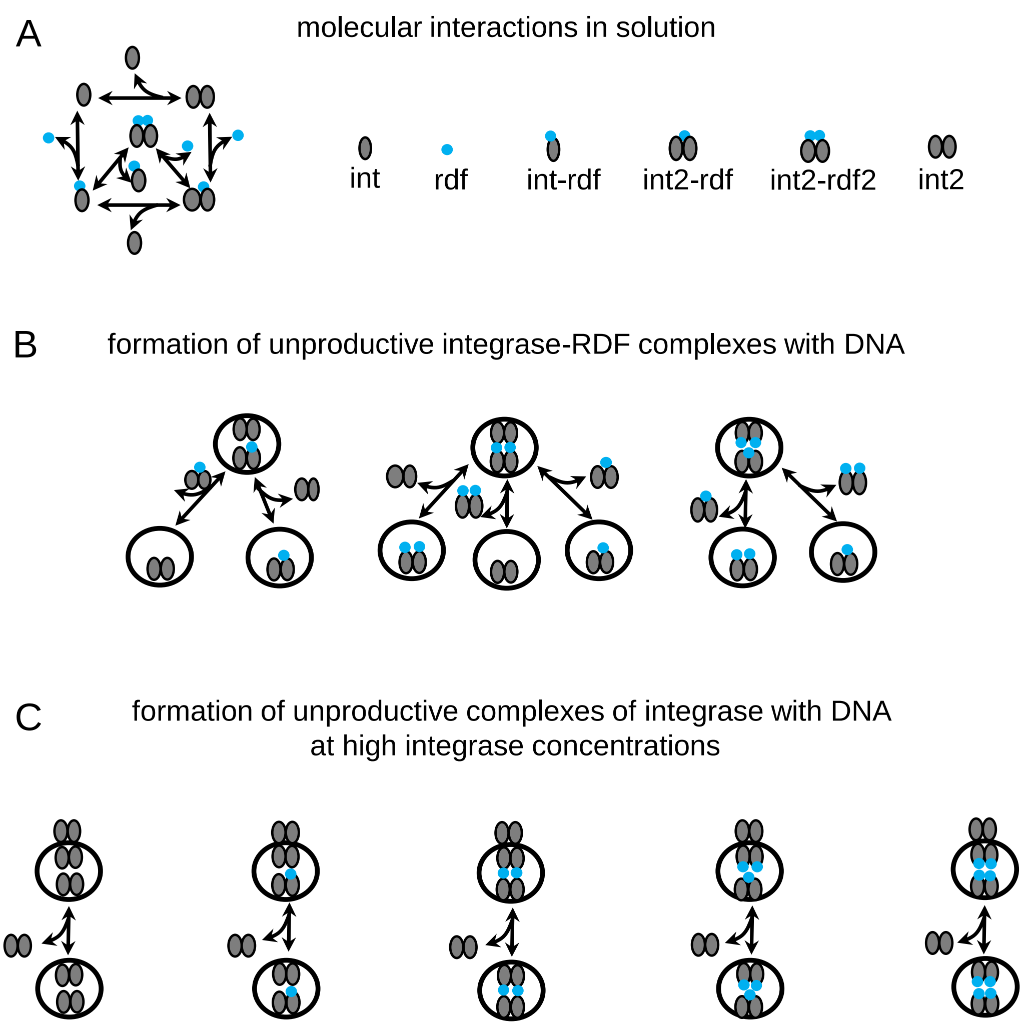

Figure S2. Schematic representation of processes involving protein-protein and protein-DNA interactions included in the models, but omitted from Fig. 1. **A**. Interactions between integrase and RDF in solution. Dimerization of integrase (with or without RDF) has dissociation constant K_{ii}. Binding of integrase complexes to RDF has dissociation constant K_{ir} (for both Model 0 and Model 1). **B**. Formation of unproductive integrase-DNA complexes with 1, 2 and 3 RDF molecules. The dissociation constants of all these unproductive complexes with pLR were assumed to be equal (K_{s01}) for simplicity. Similarly, the dissociation constants of all pPB-containing complexes with 1, 2 and 3 RDFs were assumed to be equal to K_{s02}. These interactions reduce the effective concentration of putatively productive complexes containing 4 RDF molecules (Fig. 1C) when the concentration of RDF is lower than that of integrase (for both Models 0 and 1). The substrate plasmid (pPB or pLR) is shown by an ellipse. **C**. Inhibition of recombination at high integrase concentrations in Model 1, through the formation of unproductive tetrameric complexes of integrase at single *att* sites on pPB, with equal dissociation constant (K_i) for all complexes.

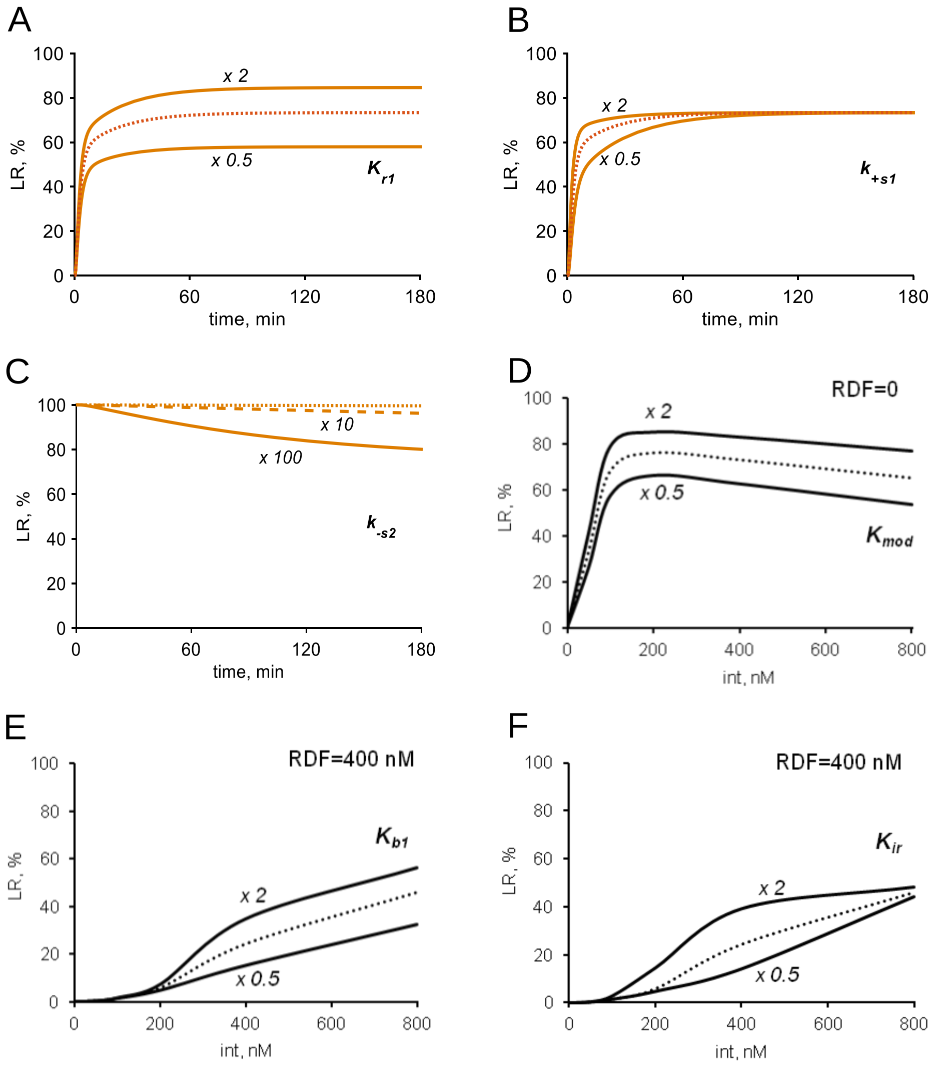

Figure S3. Typical effects of changes in the Model 1 parameters. Dotted lines show the model simulations with optimized parameters. The effects of parameters which cause the most drastic changes are illustrated (solid lines). Simulations in **A-C** were done with 400 nM integrase. **A.** Changes in the kinetics of the P × B (-RDF) reaction upon two-fold increase or decrease of the equilibrium constant of the recombination step K_{r1}. **B.** Changes in the kinetics of the P × B (-RDF) reaction upon two-fold increase or decrease of the rate constant for substrate synapse formation k_{+s1}. To keep the equilibrium constant (K_{s1}) unchanged, k_{-s1} was increased/decreased by the same amount. **C.** Changes in the kinetics of the L × R (-RDF) reaction on increasing the rate constant (k_{-s2}) of formation of the LR-int$_{s2}$ synapse. Dashed and solid lines correspond to 10- and 100-fold increases in rate constants respectively. To

keep the equilibrium constant (K_{s2}) unchanged, k_{+s2} was also increased by the same amount. **D-F.** Effect of integrase concentration on extent of P × B recombination after 3 hours. **D.** No RDF. Solid lines correspond to two-fold increase or decrease of the equilibrium constant of the modification step K_{mod}. **E.** 400 nM RDF. Solid lines correspond to two-fold increase or decrease of the equilibrium constant of the integrase binding step K_{b1}. **F.** 400 nM RDF. Solid lines correspond to two-fold increase or decrease of the equilibrium constant of the integrase-RDF binding step K_{ir}. For panels A-F, any changes in equilibrium constant were accompanied by changes in the (dependent) k_- parameter shown in the third column of supplementary table S1. All changes in equilibrium constants constrained by energy conservation (equation 36; panels A, D, E) were accompanied by compensating changes in the equilibrium constant for the slow dissociation of product synapsis (K_{s2}).

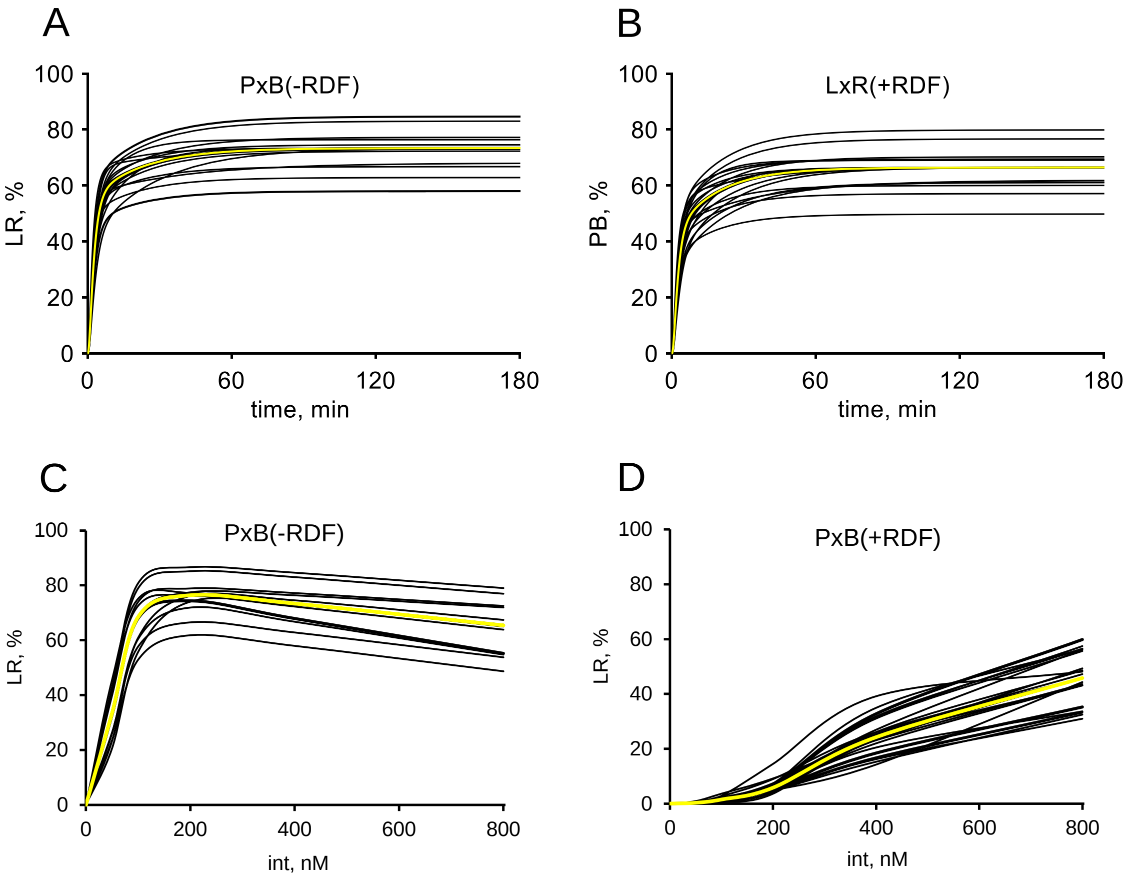

Figure S4. Robustness of the model upon 2-fold variations in the parameters. Graphs show the results of 2-fold increases and decreases of all 25 independent parameters shown in supplementary table S1. Simulations with optimized parameters are shown as yellow lines. **A.** Time courses of the P × B (-RDF) reaction (400 nM integrase). **B.** Time courses of the L × R (+RDF) reaction (400 nM integrase, 400 nM RDF). **C.** Dependence of P × B recombination after 3 hours on integrase concentration with no RDF. **D.** Dependence of P × B recombination after 3 hours on integrase concentration, with RDF at 400 nM. Changes in the equilibrium constants constrained by the energy conservation equation (36) were accompanied by compensating changes in K_{s2} or K_{s4}. All changes in independent parameters (generally in columns 1 and 2 of supplementary table S1), were accompanied by changes in the corresponding dependent parameter (generally shown in column 3 of supplementary table S1).

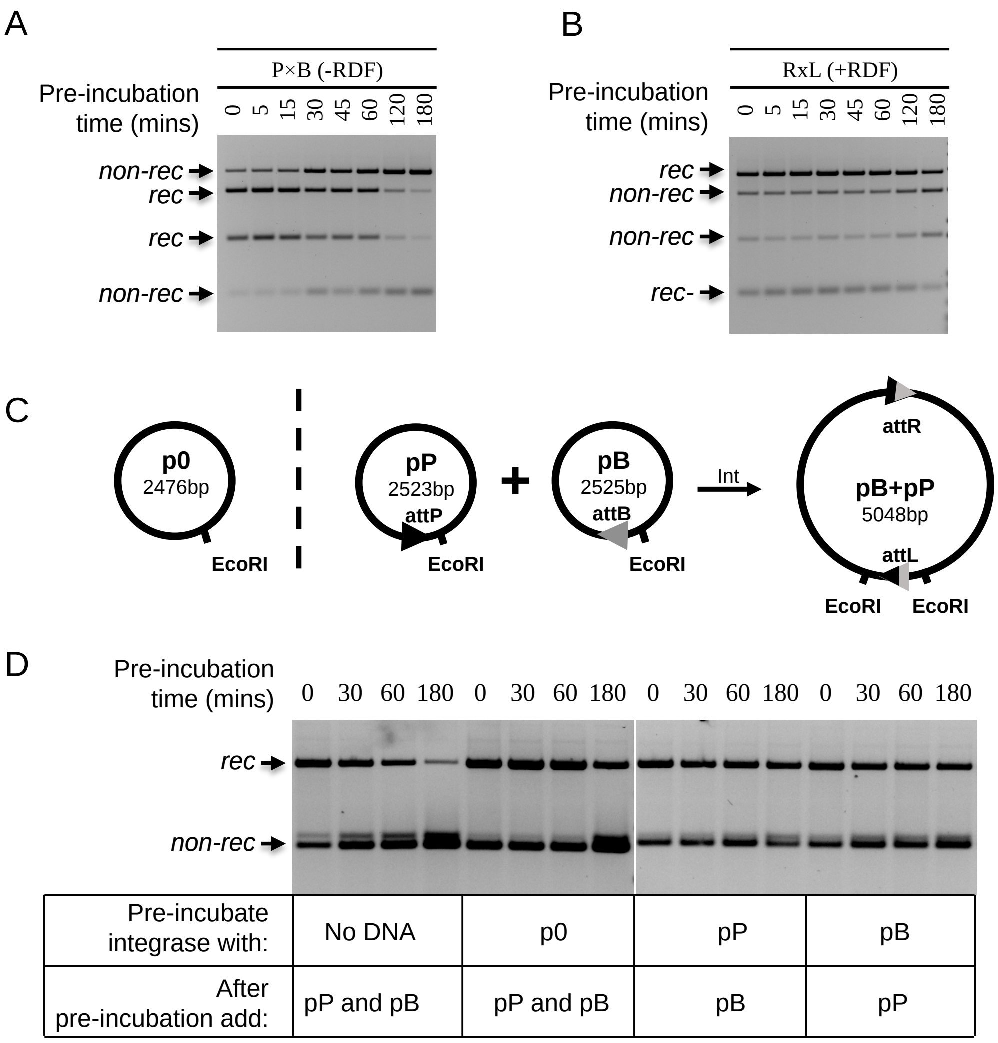

Figure S5. Effect of pre-incubation of integrase. **A.** Integrase (400 nM) was pre-incubated at 30 °C in recombination reaction buffer in the absence of DNA for the times indicated, then pPB substrate was added and the reaction was continued for 3 hours. Recombination products were analysed as in Figure 2 of the main text. non-rec, non-recombinant (substrate) bands; rec, recombinant (product) bands. **B.** As for A, except that integrase (400 nM) and RDF (800 nM) were pre-incubated together, then pLR substrate was added. **C.** Plasmids used for the intermolecular P × B recombination reaction analysed in D. Plasmid p0 lacks any *att* sites, pP contains a single *attP* site, and pB contains a single *attB* site. Recombination between pP and pB yields a 5048 bp product, which is cleaved by EcoRI to give fragments of 4987 bp and 61 bp **D.** Integrase was pre-incubated for the indicated lengths of time at 30 °C in recombination

reaction with no DNA, 10 nM non-specific DNA (p0), or 10 nM plasmid containing just *attP* or *attB* (pP or pB), as indicated below the gel. After pre-incubation, 10 nM each of pP and pB (left two panels), 10 nM pP, or 10 nM pB were added (as indicated) to initiate recombination. Reactions were then incubated for a further 3 hours at 30 °C. The products were digested with EcoRI and analysed by gel electrophoresis. Further details can be found in the supplementary methods section S1.1.2.

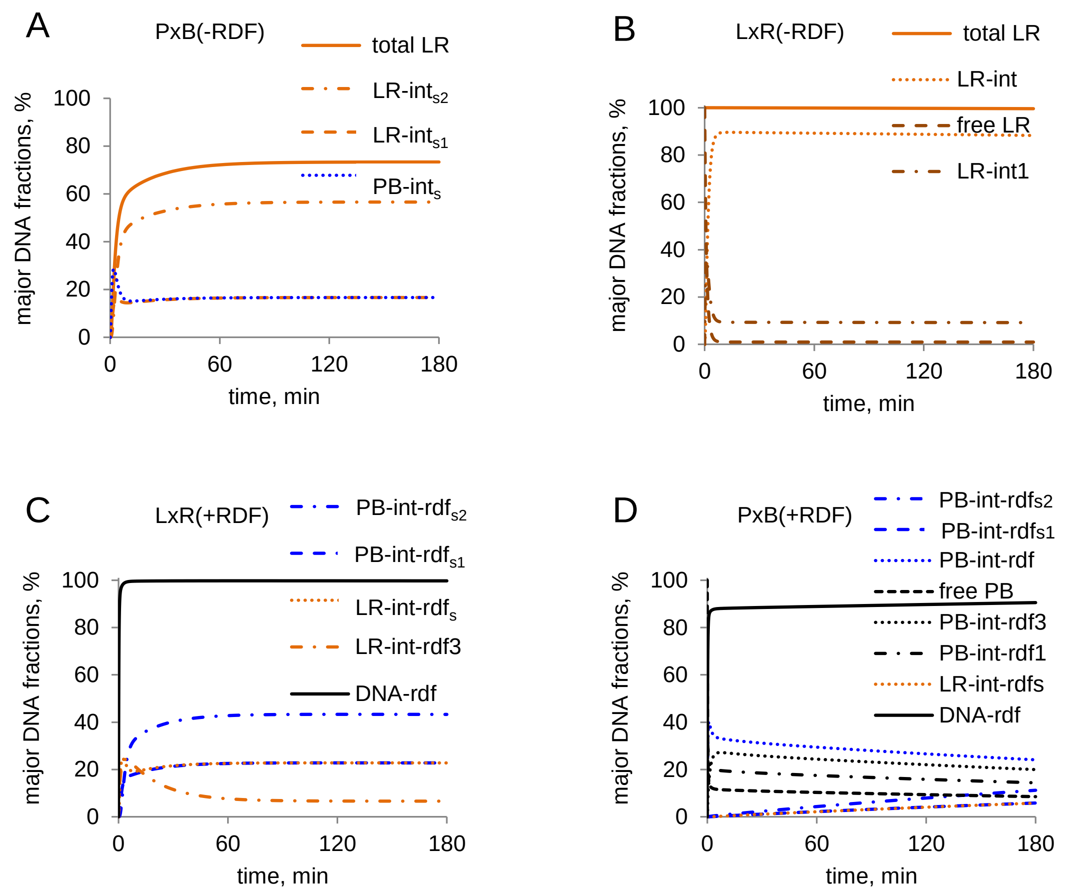

Figure S6. Simulated timecourses of all abundant DNA-containing species in Model 1. **A, B.** RDF-independent reactions; **A.** "permitted" P × B (-RDF). **B.** "non-permitted" L × R (-RDF). The graphs show the total pLR content, free pLR; pLR complexes with one (LR-int1) and two (LR-int) integrase dimers; PB and LR synapses (PB-int$_s$, LR-int$_{s1}$, LR-int$_{s2}$). **C,D.** RDF-dependent reactions; **C.** "permitted" L × R (+RDF), **D.** "non-permitted" P × B (+RDF), showing PB complexes with one (PB-int-rdf1) or two (PB-int-rdf) int2-rdf2 complexes, synapses PB-int-rdf$_{s1}$, PB-int-rdf$_{s2}$, LR-int-rdf$_s$, free PB, and PB and LR complexes with three RDF and four integrase (PB-int-rdf3 and LR-int-rdf3). In addition to LR (A,B) and PB (C,D) species (also shown in Fig. 6), which correspond to the products of the respective permitted reactions, here we show the complete set of abundant DNA species including PB (for A,B) and LR (for C,D) species. Additionally (in C,D) black lines show the total amount of RDF-containing DNA fractions, which (together with free DNA) is close to 100%. The computations were for reactions with 400 nM integrase, 10 nM plasmid substrate (pPB or pLR) and 800 nM RDF (for reactions in C and D).

S1.6 Supplementary Tables

Table S1. Parameter values of Model 1 for plasmid substrates. Designations K_{b1}, K_{b2}, K_{b3}, K_{b4}, K_{s1}, K_{s2}, K_{s3}, K_{s4}, K_r, K_{mod}, K_{modr} (shown in the upper part of the table) are equilibrium constants ($k+/k-$), with all $k+$ defined as in the clockwise direction in Fig. 1C in the main text, from pPB to pLR for the P × B (-RDF) reaction and from pLR to pPB for the L × R (+RDF) reaction. Designations K_{ii}, K_{ir}, K_i, K_{s01}, K_{s02} (shown in low part of the table) are dissociation constants for protein-protein interactions, with units µM. For steps *b1, b3, s1, s3* dissociation constants are shown in brackets for comparison with steps *b2, b4, s2, s4*.

name of step "n"	K_{eq_n}	k_{+n}	k_{-n}
b1	K_{b1}=50 µM^{-1} (K_d=0.02 µM)	k_+ = 60 µM^{-1} min^{-1}	$k_{-b1} = k_+/ K_{b1}$ = 1.2 min^{-1}
b2	K_{b2}=0.01 µM	$k_{+b2} = k_{-b2}{\cdot}K_{b2}$ = 0.1 min^{-1}	k_{-b2} = 10 µM^{-1} min^{-1}
b3	K_{b3}=40 µM^{-1} (K_d=0.025 µM)	k_+ = 60 µM^{-1} min^{-1}	$k_{-b3} = k_+/ K_{b3}$ = 1.5 min^{-1}
b4	K_{b4}=0.05 µM	$k_{+b4} = k_+{\cdot}K_{b4}$ = 3 min^{-1}	$k_{-b4} = k_+$ = 60 µM^{-1} min^{-1}
s1	K_{s1}=10 (1/K_{s1}=0.1)	k_{+s} = 0.8 min^{-1}	$k_{-s1} = k_{+s}/ K_{s1}$ = 0.08 min^{-1}
s2	K_{s2}=0.12	k_{+s2} = 0.00072 h^{-1}	$k_{-s2} = k_{+s2}/K_{s2}$ = 0.006 h^{-1}
s3	K_{s3}=10 (1/K_{s3}=0.1)	k_{+s} = 0.8 min^{-1}	$k_{-s3} = k_{+s}/ K_{s3}$ = 0.08 min^{-1}
s4	K_{s4}=0.013	k_{+s4} = 0.0039 h^{-1}	$k_{-s4} = k_{+s4}/K_{s4}$ = 0.3 h^{-1}

r	K_r=1 (K_{r1}= K_{r2})	k_{+r} = 1 min^{-1}	$k_{-r} = k_{+r}/ K_r$ = 1 min^{-1}
mod	K_{mod} =3.4	k_{+mod} = 1 min^{-1}	$k_{-mod} = k_{+mod}/ K_{mod}$=0.29 min^{-1}
modr	K_{modr} =1.9	k_{+mod} = 1 min^{-1}	k_{-modr}=k_{+mod}/ K_{modr}=0.53 min^{-1}
name of step “n”	$\boldsymbol{K_{d\_n}}$	$\boldsymbol{k_{+n}}$	$\boldsymbol{k_{-n}}$
ii	K_{ii}=0.3 µM	k_+ = 60 µM^{-1} min^{-1}	$k_{-ii} = k_+ \cdot K_{ii}$ = 18 min^{-1}
ir	K_{ir}=0.05 µM	k_+ = 60 µM^{-1} min^{-1}	$k_{-ir} = k_+ \cdot K_{ir}$ = 3 min^{-1}
i	K_i=0.02 µM	k_{+i} = 3 µM^{-1} min^{-1}	$k_{-i} = k_{+i} \cdot K_i$ = 0.06 min^{-1}
s01	K_{s01} = 0.001 µM	k_{+s0} = 40 µM^{-1} min^{-1}	$k_{-s01} = k_{+s0} \cdot K_{s01}$ = 0.04 min^{-1}
s02	K_{s02} = 0.007 µM	k_{+s0} = 40 µM^{-1} min^{-1}	$k_{-s02} = k_{+s0} \cdot K_{s02}$ = 0.28 min^{-1}

Table S2. Parameter values of Model 1 for linear DNA substrates

parameter	K_{b1}	K_{b2}	K_{b3}	K_{b4}	K_{s1}	K_{s2}	K_{s3}
value	50 μM^{-1} (K_d=0.02 μM)	0.01 μM	40 μM^{-1} (K_d=0.025 μM)	0.05 μM	303 μM^{-1} (K_d=0.0033 μM)	0.004 μM	303 μM^{-1} (K_d=0.0033 μM)
parameter	K_{s4}	K_{ii}	K_{ir}	K_r	K_{mod}	K_{modr}	K_i
value	0.00043 μM	0.3 μM	0.05 μM	1	3.4	1.9	0.05 μM
parameter	K_{s01}	K_{s02}	k_+	k_{-b2}	k_{+s0}	k_{+i}	k_{+mod}
value	0.001 μM	0.007 μM	60 μM^{-1} min^{-1}	10 μM^{-1} min^{-1}	40 μM^{-1} min^{-1}	3 μM^{-1} min^{-1}	1 min^{-1}
parameter	k_{+s}		k_{+r}	k_{-s2}		k_{-s4}	
value	24 μM^{-1} min^{-1}		0.04 min^{-1}	0.003 μM^{-1} min^{-1}		0.15 μM^{-1} min^{-1}	

Table S3. Parameter values of Model 0 for plasmid substrates

parameter	K_{b1}	K_{b2}	K_{b3}	K_{b4}	K_{s1}	K_{s2}	K_{s3}
value	50 μM^{-1} (K_d=0.02 μM)	0.0047 μM	40 μM^{-1} (K_d=0.025 μM)	0.0053 μM	10 (1/K_{s1}=0.1)	1.8	10 (1/K_{s1}=0.1)
parameter	K_{s4}	K_{ii}	K_{ir}	K_r	K_{s01}	K_{s02}	k_+
value	2.2	0.3 μM	0.05 μM	1	0.0004 μM	0.001 μM	60 μM^{-1} min^{-1}
parameter	k_{-b2}	k_{+s1}	k_{+s3}	k_{+r}	k_{+s0}		
value	10 μM^{-1} min^{-1}	0.4 min^{-1}	1 min^{-1}	1 min^{-1}	40 μM^{-1} min^{-1}		

Table S4. Experimental data. Percent recombination at different time points and in different conditions.

i) Data plotted in Figure 4A

PxB reaction, percent product yield after 3 hours
mean of 3 independent experiments (Standard deviation)

	**rdf, nM**					
**int, nM**	**0**	**50**	**100**	**200**	**400**	**800**
**0**	0% (0)	0% (0)	0% (0)	0% (0)	0% (0)	0% (0)
**50**	30% (14%)	16% (3%)	6% (1%)	4% (1%)	6% (1%)	6% (1%)
**100**	58% (19%)	47% (8%)	26% (10%)	12% (3%)	11% (1%)	12% (3%)
**200**	83% (1%)	79% (3%)	69% (11%)	31% (16%)	14% (5%)	12% (4%)
**400**	80 % (3%)	78% (4%)	73% (7%)	63% (6%)	18% (10%)	9% (2%)
**800**	67% (2%)	65% (2%)	63% (4%)	57% (5%)	45% (14%)	10% (3%)

ii) Data plotted in Figure 4B

LxR reaction, percent product yield after 3 hours
mean of 3 independent experiments (Standard deviation)

	**rdf, nM**					
**int, nM**	**0**	**50**	**100**	**200**	**400**	**800**
**0**	0% (0)	0% (0)	0% (0)	0% (0)	0% (0)	0% (0)
**50**	0% (0)	7% (5%)	13% (7%)	13% (5%)	19% (4%)	20% (4%)
**100**	0% (0)	7% (4%)	21% (9%)	34% (10%)	41% (7%)	41% (5%)
**200**	0% (0)	1% (0%)	8% (5%)	44% (8%)	68% (1%)	68% (0%)
**400**	0% (0)	1% (0%)	1% (71%)	9% (2%)	62% (4%)	67% (2%)
**800**	0% (0)	1% (0%)	1% (0%)	2% (0%)	9% (2%)	58% (2%)

iii) Data plotted in Figure 4E (orange line)

PxB reaction timecourse with 10 nM plasmids, 400 nM integrase

**Time (min)**	**% LR product**	**Standard Deviation**
0	0%	0%
1	28%	2%
2	36%	1%
4	47%	1%
8	56%	2%
16	64%	2%
32	68%	1%
64	72%	2%
120	73%	2%
180	74%	2%

iv) Data plotted in Figure 4E (blue line)

LxR reaction timemecourse with 10 nM plasmids, 400 nM integrase, 800 nM RDF

Time (min)	% PB product	Standard Deviation
0	0%	0%
1	24%	3%
2	32%	2%
4	42%	1%
8	51%	1%
16	57%	1%
32	61%	1%
64	64%	1%
120	66%	2%
180	67%	2%

v) Data plotted in Figure 7A (brown line)

PxB reaction timecourse on linear DNA, 10 nM DNA, 400 nM integrase

Time (min)	% LR product	Standard Deviation
0	0%	0%
1	1%	0%
2	2%	0%
4	4%	1%
8	7%	1%
16	12%	1%
32	19%	4%
64	25%	4%
120	32%	3%
180	37%	2%
